# Incidence and Mortality of Acute Respiratory Distress Syndrome in Patients With Burns: A Systematic Review and Meta-Analysis

**DOI:** 10.3389/fmed.2021.709642

**Published:** 2021-11-15

**Authors:** Baoli Wang, Wei Chenru, Yong Jiang, Lunyang Hu, He Fang, Feng Zhu, Qing Yu, Banghui Zhu, Guosheng Wu, Yu Sun, Zhaofan Xia

**Affiliations:** ^1^Department of Burn Surgery, The First Affiliated Hospital of Naval Medical University, Shanghai, China; ^2^Department of Burns and Plastic Surgery, General Hospital of Central Theater Command of Chinese People's Liberation Army, Shanghai, China; ^3^Research Unit of Key Techniques for Treatment of Burns and Combined Burns and Trauma Injury, Chinese Academy of Medical Sciences, Shanghai, China

**Keywords:** ARDS, incidence, mortality, burn patients, meta

## Abstract

**Objective:** We conducted a systematic review and meta-analysis to comprehensively estimate the incidence and mortality of acute respiratory distress syndrome (ARDS) in overall and subgroups of patients with burns.

**Data sources:** Pubmed, Embase, the Cochrane Library, CINAHL databases, and China National Knowledge Infrastructure database were searched until September 1, 2021.

**Study selection:** Articles that report study data on incidence or mortality of ARDS in patients with burns were selected.

**Data extraction:** Two researchers independently screened the literature, extracted data, and assessed the quality. We performed a meta-analysis of the incidence and mortality of ARDS in patients with burns using a random effects model, which made subgroup analysis according to the study type, inclusion (mechanical ventilation, minimal burn surface), definitions of ARDS, geographic location, mean age, burn severity, and inhalation injury. Primary outcomes were the incidence and mortality of burns patients with ARDS, and secondary outcomes were incidence for different subgroups.

**Data synthesis:** Pooled weighted estimate of the incidence and mortality of ARDS in patients with burns was 0.24 [95% confidence interval (CI)0.2–0.28] and 0.31 [95% CI 0.18−0.44]. Incidences of ARDS were obviously higher in patients on mechanical ventilation (incidence = 0.37), diagnosed by Berlin definition (incidence = 0.35), and with over 50% inhalation injury proportion (incidence = 0.41) than in overall patients with burns. Patients with burns who came from western countries and with inhalation injury have a significantly higher incidence of ARDS compared with those who came from Asian/African countries (0.28 vs. 0.25) and without inhalation injury (0.41 vs. 0.24).

**Conclusion:** This systematic review and meta-analysis revealed that the incidence of ARDS in patients with burns is 24% and that mortality is as high as 31%. The incidence rates are related to mechanical ventilation, location, and inhalation injury. The patients with burns from western countries and with inhalation injury have a significantly higher incidence than patients from Asian/African countries and without inhalation injury.

**Systematic Review Registration:** identifier: CRD42021144888.

## Introduction

Acute respiratory distress syndrome is very common in critically ill patients. After years of basic and clinical research, its diagnosis and treatment are improving daily, but the associated mortality rate is still as high as 30% (30-day mortality) (1). The causes of ARDS are diverse and, excluding cardiac-induced conditions, can include severe infection, shock, trauma, and burns. These injuries can induce diffuse pulmonary interstitial and alveolar edema, resulting in acute hypoxic respiratory insufficiency or failure (2). Among the causes of ARDS, severe burns can also cause a series of pathophysiological changes in various organs of the body. Among them, the lung is one of the earliest organs to be damaged, and damage to the lung is one of the main causes of death in severely burned patients (3). Although many studies have reported the incidence, treatment, and outcome of ARDS, there is no meta-analysis of ARDS in patients with burns. Our objective is to comprehensively collect published literature on ARDS in patients with burns and assess the incidence/mortality in overall and subgroups of the patient with burns.

## Methods

### Literature Search

We retrieved studies from Pubmed, Embase, the Cochrane Library, CINAHL databases, and the China National Knowledge Infrastructure database. The retrieval time for each database is from the formation of the database to September 1, 2021. We searched the databases by combining subject words and free words. Search terms included “Respiratory Distress Syndrome, Adult,” AND “incidence OR Mortality,” AND “burn.” Detailed search strategies are provided in Appendix 1. We evaluated the qualifications of the identified publications and independently extracted data from the studies selected. Differences were resolved through a consensus.

### Study Selection

Inclusion criteria were as follows: (1) study type was an observational study, case-control study, cohort study, or randomized controlled trial; (2) subjects were patients with burns; (3) incidence or mortality of ARDS was reported; (4) articles were written in English or Chinese. Exclusion criteria were as follows: (1) studies with obvious abnormal and incomplete data sets; (2) repeated publishing of the same batch of data by multiple articles; (3) sample size of the study was <20; (4) research subjects were special populations, such as children or pregnant women; (5) research subjects were only patients with inhalation injury; (6) any limitation in the length of stay and death; (7) comments, reviews, or lectures.

### Data Extraction

We extracted the characteristics of the study (author, publication year, study type, study area and centers, sample size, and study quality), basic characteristics of the research subject (average age, total body surface area (TBSA), full-thickness burn injury, inhalation injury), ARDS cases, and ARDS-related deaths. Two researchers separately collected the data and cross-checked the sampling of the other.

### Quality Assessment

Methodological quality assessment studies were also conducted by two separate researchers. We took the same type of meta-analysis “Incidence and Mortality of Acute Respiratory Distress Syndrome in Children: A Systematic Review and Meta-Analysis” as a reference, and used the evaluation tools of the research for evaluation. Our study refers to the quality evaluation in the relevant literature, and the quality of each method (bias risk) was based on a list of 13 items (4). The list of 13 items is provided in Appendix 2.

### Quantitative Data Synthesis

#### Statistical Pooling and Evaluation of Heterogeneity

We conducted a meta-analysis with the Meta package (metaprop, version R3.5.3). The data were converted with four estimation methods, and a normality test was performed on the data before the meta-analysis. In accordance with the test results, the method closest to a normal distribution was selected. Then, we combined the data (ARDS incidence and mortality) and performed a heterogeneity analysis. The confidence interval (CI) was 95%, and the statistical heterogeneity was judged by calculating *I*^2^. We choose a fixed effects model or a random effects model based on *p*-value and *I*^2^. A sensitivity analysis was performed to investigate the stability of the meta-analysis. The funnel plot method was used to judge publication bias.

#### Subgroup Analyses

We performed a subgroup meta-analysis to obtain the rate of special groups of patients with burns and explore potential sources of heterogeneity. We assessed factors, including the study type, inclusion (mechanical ventilation, minimal burn surface), definitions of ARDS, geographic location, mean age, burn severity, and inhalation injury. Divided by these factors, the combined weighted estimates were used to derive the incidence and mortality of ARDS in different subgroups. The statistical tests were all two-sided with a level of α = 0.1.

## Results

### Study Characteristics

**Figure 1:**
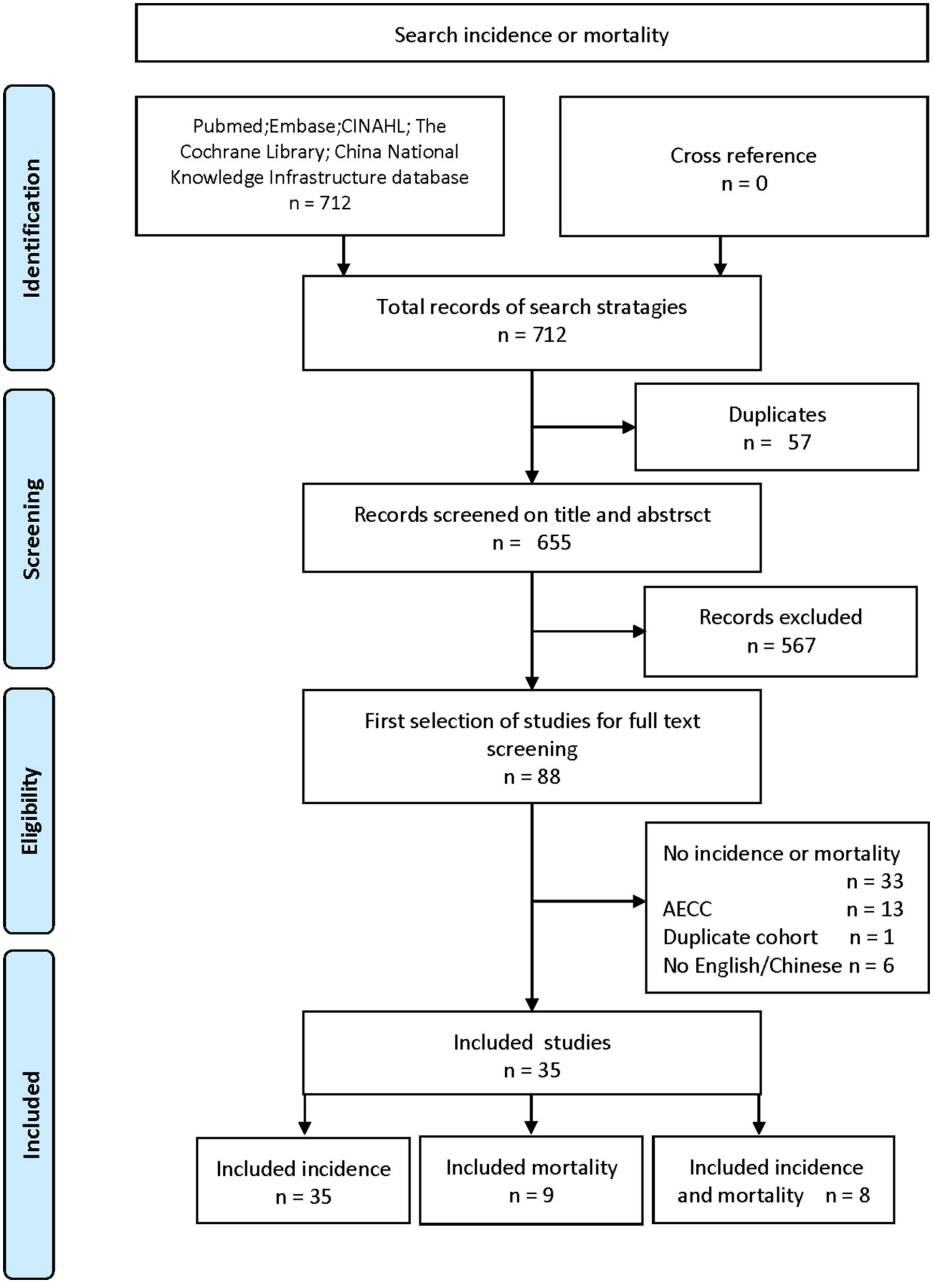
Flowchart of literature searching and inclusion.

The search identified 712 reports potentially pertaining to the morbidity and mortality of ARDS in patients with burns. After screening, 35 publications on incidence (5–39) and 9 on mortality (5, 7, 9, 11, 24, 32–34, 38) were considered to be eligible (Figure 1). The basic characteristics of the included studies, with respect to incidence and mortality, are shown in Supplementary Tables 1, 2. Most of the studies were conducted in a single center. The research population of “Incidence” was 10,899 and that of “Mortality” was 2,771. These data were from multiple countries on multiple continents; however, most of the studies were carried out in the United States, Canada, China, and Spain. The research time window of all the included studies was from 1978 to 2021. The study subjects were patients with burn, with ARDS-related records. In most of the publications, the age limit of the patients was over 18 years. In 1994, the American-European Consensus Conference (AECC) definition of ARDS was published, which had problems with lack of a criterion for acute onset, the need for a pulmonary artery catheter, and difficult hypoxemia criteria. Therefore, a new definition of ARDS, called the Berlin definition, was published, in 2012 (40). Since the definition of ARDS was constantly being adjusted with the development of clinical guidelines and practice, some studies continued to clarify the criteria used in the definition of ARDS. Most studies provide burn-related data, such as mean TBSA (%), mean full-thickness burn injury (%), and proportion with inhalation injury (%).

### Quality Assessment

The methodological quality of the study was good (average score of “Incidence” 77.1 [50–88]; the average score of “Mortality” 80.6 [69–86]). The detailed quality assessment is shown in Supplementary Tables 3, 4.

### Quantitative Data Synthesis

#### Overall Incidence and Mortality of ARDS in Patients With Burns

**Figure 2:**
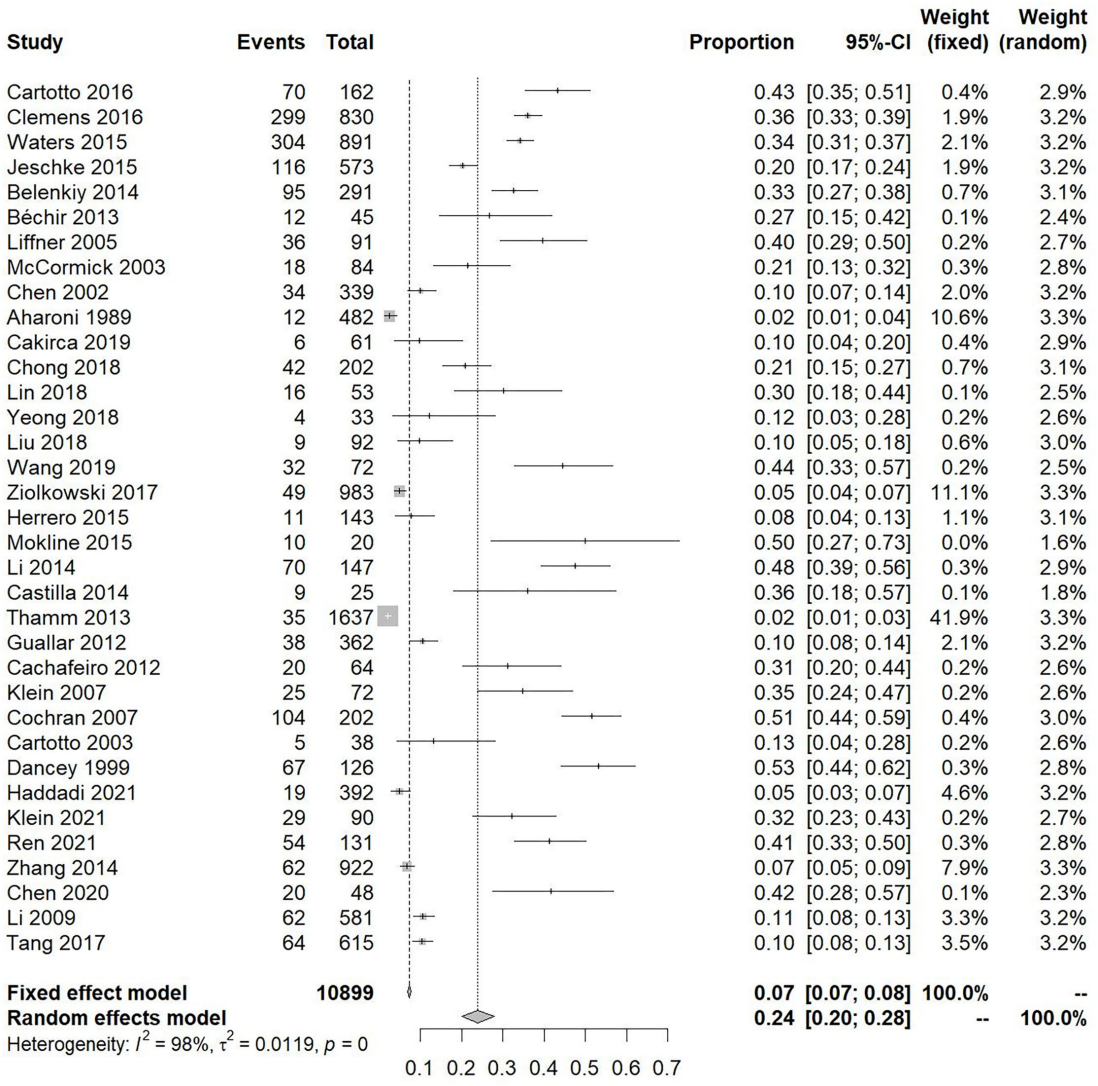
Forest plot showing the overall incidence of acute respiratory distress syndrome in patients with burns.

**Figure 3:**
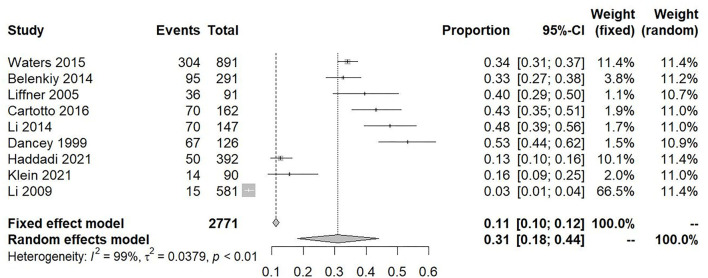
Forest plot showing the overall mortality of acute respiratory distress syndrome in patients with burns.

The incidence of pooled weighted ARDS in patients with burns was 0.24 [95% CI 0.2–0.28] (Figure 2). Patients with burns had a pooled weighted ARDS mortality of 0.31 [95% CI 0.18–0.44] (Figure 3). Studies assessing burn patient incidence (*I*^2^ = 98%) and burn patient mortality (*I*^2^ = 99%) showed significant heterogeneity. The heterogeneity of “incidence” and “mortality” is so high that they challenge the relevance of the studies, and they could come from the differences in the inclusion criteria of patients (such as mechanical ventilation and minimal burn surface), definitions of ARDS, geographic location, mean age, burn severity, and inhalation injury, so we made the subgroup analysis to get the accurate incidence of special burn patients. Due to the number of studies included in mortality analysis being only 9, we did not divide these studies into subgroups.

#### Incidence of ARDS in Patients With Burns by Different Study Type

Considering the type of study that may influence the incidence of ARDS in patients with burns, we divided the studies into two types (retrospective and prospective). There are 15 retrospective studies and 3 prospective studies; the “retrospective” subgroup has 6,685 patients in total, and the “prospective” subgroup has 245 patients. For patients with burns in the retrospective studies, the incidence of ARDS was 0.24 [95% CI 0.18–0.3], while that for burns patients in the retrospective studies, the incidence of 0.15 [95% CI 0.06–0.39]. *I*^2^ was 99 and 86%, respectively. No statistical difference was noted between them (*p* = 0.33) (Supplementary Figure 1; Table 1).

**Table 1:** Pooled estimation of incidence of acute respiratory distress syndrome in burn patients and its subgroup.

**Factors**	**Subgroups**	**Studies, *n***	**No. of patients**	**Proportion [95% CI]^**a**^**	***I* ^2^ ^**b**^**	***P*-value for heterogeneity**	***P*-value for subgroup differences**
Study type	Retrospective	15	6,685	0.24 [0.18–0.30]	99%	<0.01	0.33
	Prospective	3	245	0.15 [0.06–0.39]	86%	<0.01	
Inclusion	With mechanical ventilation	8	2,630	0.37 [0.29–0.44]	93%	<0.01	–^g^
	minima burn surface ≥20%	9	1,127	0.32 [0.21–0.42]	92%	<0.01	–
Definition^c^	Berlin	9	2,738	0.35 [0.31–0.40]	81%	<0.01	0.61
	AECC	6	1,989	0.30 [0.08–0.51]	98%	<0.01	
Location	Western	22	9,100	0.28 [0.27–0.33]	99%	<0.01	**0.01**
	Asian/African	13	12,659	0.25 [0.22–0.29]	95%	<0.01	
Mean age	20y−39y	10	3,097	0.25 [0.14–0.36]	98%	<0.01	0.73
	40y−59y	18	7,542	0.27 [0.21–0.33]	97%	<0.01	
TBSA^d^	<30%	9	4,763	0.22 [0.15–0.29]	99%	<0.01	0.22
	≥30%	21	3,877	0.28 [0.22–0.34]	95%	<0.01	
FT^e^	≤ 10%	3	970	0.29 [0.09–0.49]	97%	<0.01	0.91
	>10%	15	5,691	0.30 [0.23–0.37]	99%	<0.01	
II^f^	<50%	18	6,909	0.24 [0.17–0.30]	99%	<0.01	** <0.01**
	≥50%	4	387	0.41 [0.34–0.48]	49%	0.12	

*^(a)^ 95% CI: 95% confidence interval*.

*^(b)^ I^2^: heterogeneity of the studies*.

*^(c)^ Definition: which definition of acute respiratory distress syndrome used in the studies, the American-European Consensus Conference (AECC) definition or the Berlin definition*.

*^(d)^ TBSA, total body surface area*.

*^(e)^ FT, full-thickness (FT) burn injury*.

*^(f)^ II: the proportion of patients combined inhalation injury of the total sample*.

*^(g)^ “–” means the date is not available*.

*Bold values mean statistically significant*.

#### Incidence of ARDS in Patients With Burns and on Mechanical Ventilation

We found a total of eight studies containing mechanical ventilation in the inclusion criteria. The number of patients with burns and mechanical ventilation was 2,630, and for these patients, the incidence of ARDS was 0.37 [95% CI 0.29–0.44], and the heterogeneity was decreased to 93% (Supplementary Figure 2; Table 1).

#### Incidence of ARDS in Patients With Burns and TBSA ≥20%

Some “incidence” studies used TBSA (%) as an inclusion criterion, which included TBSA ≥1, 20, 30%, and so on. There were nine studies that set TBSA to ≥20% as an inclusion criterion, two studies set TBSA to ≥30%, and one study set TBSA to ≥50%. The minima burn surfaces of all these patients (*n* = 1127) were over 20%. The incidence of ARDS in these patients was 0.32 [95% CI 0.21–0.42], and heterogeneity was 92% (Supplementary Figure 3; Table 1).

#### Incidence of ARDS Defined by AECC and Berlin Definition

We found nine studies that defined ARDS by the Berlin definition and six studies by the AECC definition, and the number of patients was 2,738 and 1,989, respectively. The incidence of ARDS defined by the Berlin definition was 0.35, and the incidence of ARDS defined by the AECC definition (41) was 0.3 (Supplementary Figure 4). There was no significant difference between these subgroups (*p* = 0.61; Table 1).

#### Incidence of ARDS in Patients With Burns by Location

We divided the studies into Western research projects and Asian-African groups based on geography. The incidence of ARDS in burn patients in Western countries was 0.28 ([95% CI 0.27–0.33], *I*^2^ = 99%, *N* = 22), and the incidence in Asian and African countries was 0.25 ([95% CI 0.22–0.29], *I*^2^ = 95%, *N* = 13) (Supplementary Figure 5). There was no difference between the subgroups (*P* = 0.38; Table 1).

#### Incidence of ARDS in Patients With Burns by Mean Age

For studies where the average age of patients was 20–39 years, we combined the incidence of ARDS, and the outcome was 0.25 [95% CI 0.14–0.36]. For the subgroups with ages of 40–59 years, the incidence was 0.27 [95% CI 0.21–0.33] (Supplementary Figure 6). No statistical difference was found in this comparison (*P* = 0.73; Table 1).

#### Incidence of ARDS in Patients With Burns by Mean TBSA (%)

The incidence of ARDS in the TBSA ≥ 30% burn group is a little bit higher than the TBSA <30% (Table 1). The results were mean TBSA ≥ 30%,0.26 [95% CI 0.21–0.31], *N* = 21, *I*^2^ = 95% vs. mean TBSA <30%0.22 [95% CI 0.15–0.29], *N* = 9, *I*^2^ = 99% (Supplementary Figure 7).

#### Incidence of ARDS in Patients With Burns by Average Full-Thickness (%)

The incidence of ARDS in patients with burns and an average full-thickness of over 10% was 0.24 ([95% CI 0.17–0.35], N = 14, *I*^2^ = 98%). Those with an average full-thickness ≤ 10% had an incidence of 0.19 ([95% CI 0.05–0.7], N = 2, *I*^2^ = 94%; Supplementary Figure 8). The incidence was not significantly different between these groups (*P* = 0.74; Table 1).

#### Incidence of ARDS in Patients With Burns by Inhalation Injury Proportion (%)

The incidence rate in the subgroup with over 50%inhalation injury proportion in patients with burns were 0.41 ([95% CI 0.34–0.48], *N* = 4, *I*^2^ = 49%), which is significantly higher than that of the subgroup with <50% inhalation injury proportion (0.24 [95% CI 0.17–0.3], *N* = 18, *I*^2^ = 99% (Supplementary Figure 9). There was a significant difference between these subgroups (*p* <0.01; Table 1).

#### Publication Bias and Sensitivity Analysis

We used a funnel plot to test for publication bias in 35 incidence and 9 mortality studies. The inverted funnel plot suggested there was a little bit of bias in studies reporting incidence and no obvious publication bias in mortality (Supplementary Figures 10, 11). To assess whether the pooled incidence or mortality of ARDS in this meta-analysis was stable, a sensitivity analysis was performed. The effect estimation of sensitivity analysis showed that regardless of pooled incidence or pooled mortality, the results were stable (Supplementary Figures 12, 13).

## Discussion

This systematic review and meta-analysis included the first large-scale analysis of the incidence and mortality rates of ARDS in patients with burns. We calculated the pooled incidence and mortality of ARDS in patients with burns, as well as those in subgroups with alternative definitions, average age, TBSA, full-thickness burn injury, and inhalation injury proportion. These findings can help guide clinicians in assessing and diagnosing ARDS in patients with burns in the future and play an important role in the allocation of medical resources for disease and its prevention.

We selected several influencing factors related to ARDS which are common in most studies on the condition. These were used in the subgroup analysis and included study type, mechanical ventilation, minima burn surface within inclusion criteria, ARDS definition, geographic location, age, TBSA, full-thickness burn injury, and inhalation injury. We compared the rates and found that the “prospective-study type” might prevent ARDS from happening (incidence = 0.15), which may be caused by different types of treatment and healthcare. The incidences of ARDS were obviously higher in patients who is with mechanical ventilation (incidence = 0.37), whose minima burn surface were over 20% (incidence = 0.32), and which subgroup is with over 50% inhalation injury proportion (incidence = 0.41) than the common burn patients (incidence = 0.24). What is more, *I*^2^ decreases while the rate increases, which makes the incidence more credible. In our result, mechanical ventilation, location, and inhalation injury were, again, identified as risk factors of ARDS.

We found that the more severe the inhalation injury, the higher the incidence, which is in line with the majority of previous research conclusions. The patients with burns and inhalation injury have a significantly higher incidence of ARDS compared with those without inhalation injury. As such, it can be concluded that inhalation injury was an independent risk factor for ARDS. However, using the new Berlin definition of ARDS (incidence = 0.35), it was clear the rates were higher than when using the older AECC definition (incidence = 0.30). From the perspective of diagnostic criteria, The PaO_2_/FiO_2_ requirements of Berlin are higher (40). The reason why the incidence of Berlin is higher than that of AECC is that the level of medical treatment has improved significantly with the development of time. The development of sophisticated testing equipment has enabled physicians to discover more patients with potential ARDS. The result shows that ARDS was more common in the subgroup of burns patients aged 40–59 years (incidence = 0.27) than the subgroup of burns patients aged 20–39 years (incidence = 0.25). However, the number of 40–59 years group studies (*N* = 18, *n* = 7,542) is higher than 20–39 years group (*N* = 10, *n* = 3,970), which means burns are common in 20–39 years group, but ARDS was common in 40–59 years group. Although the results are surprising, they should not be a problem. We can design a large targeted prospective study to prove this result further.

As this article is a meta-analysis of a single-group rate, we encountered the common problem of high heterogeneity. The *I*^2^ of incidence was > 80%, regardless of whether heterogeneity was calculated for the “incidence” rates or the “mortality” rates. As a result, we chose to use a random effects model in the analysis. We explored the possible sources of heterogeneity in the studies of incidence by subgroup meta-analysis. Although we tried a lot of factors that may cause high levels of heterogeneity, including study type, inclusion, definition, location, mean age, TBSA, etc., we could not find a single model to account for these factors together. In the series of “incidence” studies, except for the “study style- prospective,” “definition- Berlin” subgroups, and “inhalation injury-≥50%” whose heterogeneity was slightly reduced (*I*^2^ <90), the remaining studies were more than 90% heterogeneous, with the vast majority being over 95%. When we compared the *I*^2^ with other “a single-group rate” meta-analysis (28, 42), we found that high heterogeneity was common. Therefore, it might be acceptable that *I*^2^ was 99% for the “mortality” study. For the subgroup of patients with burns and over 50% inhalation injury proportion, the incidence of ARDS (0.41) was precise because *I*^2^ = 49%. In addition to the heterogeneous sources suggested by the statistical results, we speculate that the inclusion criteria for each study could also be a main source of heterogeneity. We included diversiform literature, which were recording data related to ARDS and “burns.” Patients with burns in some of the studies had different therapeutic schedules. Besides, some studies only analyzed mechanically ventilated patients with burns. The funnel plots showed that the distribution of “incidence” was asymmetric. The bias was very strong (*p* = 0.01). These variations in methodology and patient sampling may be the reason for the high heterogeneity. The distribution for “mortality” was visually symmetrical based on the results of the funnel plot. Furthermore, after sensitivity testing, the aggregated weighted ARDS morbidity and mortality were stable.

This meta-analysis has several advantages. First, this is the first comprehensive systematic analysis of ARDS in patients with burns. Most of the previous studies on the subject were based on specific groups (large area burns, burns with inhalation injuries, etc.). Comparing the findings here with previous studies, we note that this study is more thorough in including overall types of patients with burns. This leads to a more comprehensive and accurate assessment of the incidence and mortality rates of ARDS. Second, we use a rigorous screening method to exclude studies from different subject areas conducted by the same authors, while conducting detailed quality assessments of the articles included. Third, we made the most of the more than 8,000 samples; refining the study population classification according to different indicators, obtaining the incidence rates of different subgroups, and determining the relevant risk factors for ARDS. However, there are also some limitations in our research. First, our target population includes overall patients with burns. While the sensitivity analysis shows that the incidence and mortality rates are stable, we do recognize that there are many influencing factors, such as mechanical ventilation, definition, geographic location, inclusion criteria, age, burn severity, and inhalation injury in ARDS. The influence of all these various factors causes a substantial amount of heterogeneity in the data. Second, we were unable to retrieve data on individual patients and only conducted a meta-analysis on the results of each study. We used these data to determine the risk factors of ARDS based on the rate of events and characteristics of the study population. In subsequent studies, it would be advantageous to arrange a large and multicenter cohort of burn victims to address these issues.

## Conclusion

This systematic review and meta-analysis revealed that the incidence of ARDS in patients with burns is 24% and that mortality is as high as 31%. The incidence rates are related to mechanical ventilation, location, and inhalation injury. Patients with burns from western countries and with inhalation injury have a significantly higher incidence than patients from Asian/African countries and without inhalation injury.

## Data Availability Statement

The original contributions presented in the study are included in the article/Supplementary Material, further inquiries can be directed to the corresponding author/s.

## Author Contributions

BW, ZX, YS, and GW: substantial contributions to the conception and design of the study. BW, WC, YJ, LH, and GW: acquisition, analysis, and interpretation of data for the study. BW, WC, YJ, HF, FZ, QY, and BZ: drafting of the article. BW, GW, YS, and ZX: revising the article critically for important intellectual content. GW, YS, and ZX: final approval of the version submitted for publication. All authors contributed to the article and approved the submitted version.

## Funding

This study was funded by CAMS Innovation Fund for Medical Sciences (2019-I2M-5-076), the National Natural Science Foundation of China (81772125), and Shanghai Sailing Program (18YF1422900).

## Conflict of Interest

The authors declare that the research was conducted in the absence of any commercial or financial relationships that could be construed as a potential conflict of interest.

## Publisher's Note

All claims expressed in this article are solely those of the authors and do not necessarily represent those of their affiliated organizations, or those of the publisher, the editors and the reviewers. Any product that may be evaluated in this article, or claim that may be made by its manufacturer, is not guaranteed or endorsed by the publisher.

## Supplementary Material

Supplementary Figure 1 Forest plot: incidence for the retrospective and prospective subgroups.

Supplementary Figure 2 Forest plot showing the incidence of acute respiratory distress syndrome in patients with burns and on mechanical ventilation.

Supplementary Figure 3 Forest plot showing the incidence of acute respiratory distress syndrome in patients with burn and TBSA ≥ 20%.

Supplementary Figure 4 Forest plot: incidence for the subgroups of American-European Consensus Conference definition and Berlin definition.

Supplementary Figure 5 Forest plot: incidence for the subgroups of Western countries and Asian and African countries.

Supplementary Figure 6 Forest plot: incidence for the subgroups of 20–39- and 40−59-year-olds.

Supplementary Figure 7 Forest plot: incidence for the subgroups of mean total body surface area <30 and ≥30%.

Supplementary Figure 8 Forest plot: incidence for the subgroups of inclusion of full-thickness burn injury ≤ 10 and >10%.

Supplementary Figure 9 Forest plot: incidence for the subgroups of patients with combined inhalation injury <50 and ≥50%.

Supplementary Figure 10 Funnel plot of incidence studies.

Supplementary Figure 11 Funnel plot of mortality studies.

Supplementary Figure 12 Sensitive analysis for incidence of acute respiratory distress syndrome in patients with burns.

Supplementary Figure 13 Sensitive analysis for the mortality of acute respiratory distress syndrome in patients with burns.

Supplementary Table 1 Characteristic of studies included in meta-analysis of incidence in burn patients.

Supplementary Table 2 Characteristic of studies included in meta-analysis of mortality in burn patients.

Supplementary Table 3 Quality assessment of the included studies on incidence of acute respiratory distress syndrome in burn patient with full-text.

Supplementary Table 4 Quality assessment of the included studies on mortality of acute respiratory distress syndrome in burn patient with full-text.

Appendix 1 Search strategies of PubMed, Embase, CINAHL databases, and China National Knowledge Infrastructure database.

Appendix 2 Quality assessment of the included studies.

## Abbreviations

ARDS: acute respiratory distress syndrome
TBSA: total body surface area
AECC: American-European Consensus Conference.

## References

[1] Shaw TD McAuley DF O'Kane CM. Emerging drugs for treating the acute respiratory distress syndrome. Expert Opin Emerg Drugs. (2019) 24:29–41. 10.1080/14728214.2019.1591369 30841764

[2] Bittner EA Shank E Woodson L Martyn JA. Acute and perioperative care of the burn-injured patient. Anesthesiology. (2015) 122:448–64. 10.1097/ALN.0000000000000559 25485468 PMC4844008

[3] Nielson CB Duethman NC Howard JM Moncure M Wood JG. Burns: pathophysiology of systemic complications and current management. J Burn Care Res. (2017) 38:e469–81. 10.1097/BCR.0000000000000355 27183443 PMC5214064

[4] Schouten LR Veltkamp F Bos AP van Woensel JB Serpa Neto A Schultz MJ . Incidence and mortality of acute respiratory distress syndrome in children: a systematic review and meta-analysis. Crit Care Med. (2016) 44:819–29. 10.1097/CCM.0000000000001388 26509320

[5] Cartotto R Li Z Hanna S Spano S Wood D Chung K . The Acute Respiratory Distress Syndrome (ARDS) in mechanically ventilated burn patients: an analysis of risk factors, clinical features, and outcomes using the Berlin ARDS definition. Burns. (2016) 42:1423–32. 10.1016/j.burns.2016.01.031 27520712

[6] Clemens MS Stewart IJ Sosnov JA Howard JT Belenkiy SM Sine CR . Reciprocal risk of acute kidney injury and acute respiratory distress syndrome in critically ill burn patients. Crit Care Med. (2016) 44:e915–22. 10.1097/CCM.0000000000001812 27340755

[7] Waters JA Lundy JB Aden JK Sine CR Buel AR Henderson JL . A comparison of acute respiratory distress syndrome outcomes between military and civilian burn patients. Mil Med. (2015) 180:56–9. 10.7205/MILMED-D-14-00390 25747632

[8] Jeschke MG Pinto R Kraft R Nathens AB Finnerty CC Gamelli RL . Morbidity and survival probability in burn patients in modern burn care. Crit Care Med. (2015) 43:808–15. 10.1097/CCM.0000000000000790 25559438 PMC4359665

[9] Belenkiy SM Buel AR Cannon JW Sine CR Aden JK Henderson JL . Acute respiratory distress syndrome in wartime military burns: application of the Berlin criteria. J Trauma Acute Care Surg. (2014) 76:821–7. 10.1097/TA.0b013e3182aa2d21 24553555

[10] Bechir M Puhan MA Fasshauer M Schuepbach RA Stocker R Neff TA. Early fluid resuscitation with hydroxyethyl starch 130/0.4 (6%) in severe burn injury: a randomized, controlled, double-blind clinical trial. Crit Care. (2013) 17:R299. 10.1186/cc13168 24365167 PMC4057504

[11] Liffner G Bak Z Reske A Sjoberg F. Inhalation injury assessed by score does not contribute to the development of acute respiratory distress syndrome in burn victims. Burns. (2005) 31:263–8. 10.1016/j.burns.2004.11.003 15774279

[12] McCormick JT O'Mara MS Wakefield W Goldfarb IW Slater H Caushaj PF. Effect of diagnosis and treatment of sinusitis in critically ill burn victims. Burns. (2003) 29:79–81. 10.1016/S0305-4179(02)00233-4 12543050

[13] Chen XL Wang YJ Wang CR Hu DL Sun YX Li SS. Burns due to gunpowder explosions in fireworks factory: a 13-year retrospective study. Burns. (2002) 28:245–9. 10.1016/S0305-4179(01)00122-X 11996855

[14] Aharoni A Moscona R Kremerman S Paltieli Y Hirshowitz B. Pulmonary complications in burn patients resuscitated with a low-volume colloid solution. Burns. (1989) 15:281–4. 10.1016/0305-4179(89)90001-6 2590399

[15] Cakirca M Sozen I Tozlu Bindal G Baydar M Yasti AC. Relationship between the albumin level and the anesthesia method and the effect on clinical course in patients with major burns. Ulus Travma Acil Cerrahi Derg. (2019) 25:55–9. 10.5505/tjtes.2018.71278 30742287

[16] Chong SJ Kok YO Tay RXY Ramesh DS Tan KC Tan BK. Quantifying the impact of inhalational burns: a prospective study. Burns Trauma. (2018) 6:26. 10.1186/s41038-018-0126-z 30238012 PMC6139897

[17] Lin KH Chu CM Lin YK Chiao HY Pu TW Tsai YM . The abbreviated burn severity index as a predictor of acute respiratory distress syndrome in young individuals with severe flammable starch-based powder burn. Burns. (2018) 44:1573–8. 10.1016/j.burns.2018.01.006 29886117

[18] Yeong EK O'Boyle CP Huang HF Tai HC Hsu YC Chuang SY . Response of a local hospital to a burn disaster: contributory factors leading to zero mortality outcomes. Burns. (2018) 44:1083–90. 10.1016/j.burns.2018.03.019 29753454

[19] Liu K Wood D Cartotto R. Mechanical ventilation of burn patients who do not have the acute respiratory distress syndrome (ARDS). J Burn Care Res. (2018) 39:S68. 10.1093/jbcr/iry006.127

[20] Wang W Yu X Zuo F Yu S Luo Z Liu J . Risk factors and the associated limit values for abnormal elevation of extravascular lung water in severely burned adults. Burns. (2019) 45:849–59. 10.1016/j.burns.2018.11.007 30527647

[21] Ziolkowski N Rogers AD Xiong W Hong B Patel S Trull B . The impact of operative time and hypothermia in acute burn surgery. Burns. (2017) 43:1673–81. 10.1016/j.burns.2017.10.001 29089204 PMC7865205

[22] Herrero EH Sánchez M Cachafeiro L Agrifoglio A Galván B Asensio MJ . Lactate in the burn patient. Crit Care. (2015) 19:S50. 10.1186/cc14225

[23] Mokline A Rahmani I Gharsallah L Hachani A Tlaili S Hammouda R . Intraabdominal hypertension in burn patients. Crit Care. (2015) 19:S136. 10.1186/cc14467

[24] Li Z Chung K Cartotto R. Application of the new berlin definition of Acute Respiratory Distress Syndrome (ARDS) to burn patients. J Burn Care Res. (2014) 35:S65. 10.1097/01.bcr.0000445188.61189.d4 24642717

[25] Ruiz-Castilla M Barret JP Sanz D Aguilera J Serracanta J Garcia V . Analysis of intra-abdominal hypertension in severe burned patients: the Vall d'Hebron experience. Burns. (2014) 40:719–24. 10.1016/j.burns.2013.09.021 24199890

[26] Thamm OC Perbix W Zinser MJ Koenen P Wafaisade A Maegele M . Early single-shot intravenous steroids do not affect pulmonary complications and mortality in burned or scalded patients. Burns. (2013) 39:935–41. 10.1016/j.burns.2012.10.007 23146575

[27] GuallarC SaénchezM CachafeiroL HerreroE AsensioMJ HernandezM . An epidemiologic study of burn patients admitted in a burn intensive care unit. Intensive Care Med. (2012) 38:S84–5. 10.1007/s00134-012-2683-023064528

[28] Cachafeiro L Sanchez M Herrero E Camacho J Hernandez M Agrifoglio A . Epidemiological study of critical burn patients in an ICU. Crit Care. (2012) 16:S164–5. 10.1186/cc11070

[29] Klein MB Hayden D Elson C Nathens AB Gamelli RL Gibran NS . The association between fluid administration and outcome following major burn: a multicenter study. Ann Surg. (2007) 245:622–8. 10.1097/01.sla.0000252572.50684.49 17414612 PMC1877030

[30] Cochran A Morris SE Edelman LS Saffle JR. Burn patient characteristics and outcomes following resuscitation with albumin. Burns. (2007) 33:25–30. 10.1016/j.burns.2006.10.005 17223485

[31] Cartotto R Choi J Gomez M Cooper A. A prospective study on the implications of a base deficit during fluid resuscitation. J Burn Care Rehabil. (2003) 24:75–84. 10.1097/01.BCR.0000054177.24411.13 12626925

[32] Dancey DR Hayes J Gomez M Schouten D Fish J Peters W . ARDS in patients with thermal injury. Intensive Care Med. (1999) 25:1231–6. 10.1007/PL00003763 10654206

[33] Haddadi S Parvizi A Niknama R Nemati S Farzan R Kazemnejad E. Baseline characteristics and outcomes of patients with head and neck burn injuries; a cross-sectional study of 2181 cases. Arch Acad Emerg Med. (2021) 9:e8. 10.22037/aem.v9i1.948 33490965 PMC7812157

[34] Klein HJ Rittirsch D Buehler PK Schweizer R Giovanoli P Cinelli P . Response of routine inflammatory biomarkers and novel Pancreatic Stone Protein to inhalation injury and its interference with sepsis detection in severely burned patients. Burns. (2021) 47:338–48. 10.1016/j.burns.2020.04.039 33272743

[35] Ren HT Chen HQ Han CM. Establishment of a predictive model for acute respiratory distress syndrome and analysis of its predictive value in critical burn patients. Zhonghua Shao Shang Za Zhi. (2021) 37:333–9. 10.3760/cma.j.cn501120-20200301-00109 33745255 PMC11917325

[36] Zhang T Li X Deng Z Zhang Z Tang W Chen B . Analysis of respiratory complications in 922 severely burned patients. Zhonghua Shao Shang Za Zhi. (2014) 30:199–202. 25174379

[37] Chen JY Liu SX. Influence of directed restrictive fluid management strategy on patients with severe burns complicated by inhalation injury. Zhongguo Shaoshang Chuangyang Za Zi. (2020) 32:340–51. Available online at: https://kns.cnki.net/kcms/detail/detail.aspx?FileName=SSCS202005010&DbName=CJFQ202031357819

[38] Li XJ Zhang Z Huo LZ Liang DR Liang R Zhong XM . Respiratory system complications and treatment of severe burns patients. China Acad J Electr Publ House. (2009) 1:283–4. Available online at: https://kns.cnki.net/kcms/detail/detail.aspx?FileName=SSWK200911001511&DbName=CPFD2010

[39] Tang Y Wang LX Xie WG Shen ZA Guo GH Chen JJ . [Multicenter epidemiological investigation of hospitalized elderly, young and middle-aged patients with severe burn]. Zhonghua Shao Shang Za Zhi. (2017) 33:537–44. 10.3760/cma.j.issn.1009-2587.2017.09.003 28926874

[40] Lam NN Hung TD Hung DK. Acute respiratory distress syndrome among severe burn patients in a developing country: application result of the berlin definition. Ann Burns Fire Disasters. (2018) 31:9–12. 30174564 PMC6116651

[41] Sine CR Belenkiy SM Buel AR Waters JA Lundy JB Henderson JL . Acute respiratory distress syndrome in burn patients: a comparison of the berlin and american-european definitions. J Burn Care Res. (2016) 37:e461–9. 10.1097/BCR.0000000000000348 27070223

[42] Hirsch L Jette N Frolkis A Steeves T Pringsheim T. The incidence of parkinson's disease: a systematic review and meta-analysis. Neuroepidemiology. (2016) 46:292–300. 10.1159/000445751 27105081

