# Supplementary figures and images for: Incidence and Mortality of Acute Respiratory Distress Syndrome in Patients With Burns: A Systematic Review and Meta-Analysis
Source: Front Med (Lausanne). 2021 Nov 15;8:709642. doi: 10.3389/fmed.2021.709642 (PMC8634659; doi:10.3389/fmed.2021.709642)

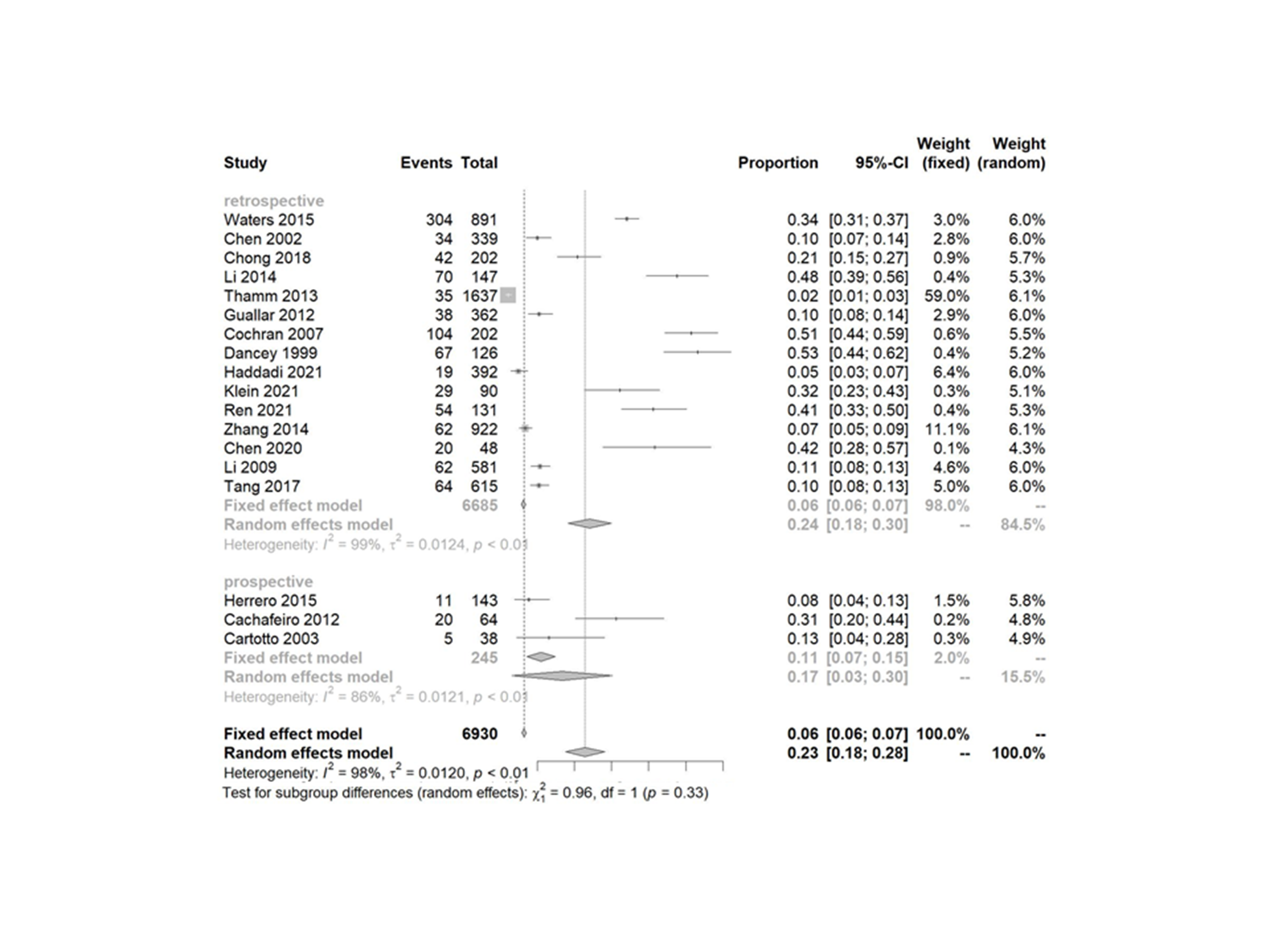

Supplement: Supplementary Figure 1 — Forest plot: incidence for the retrospective and prospective subgroups. [file Image_1.TIF]

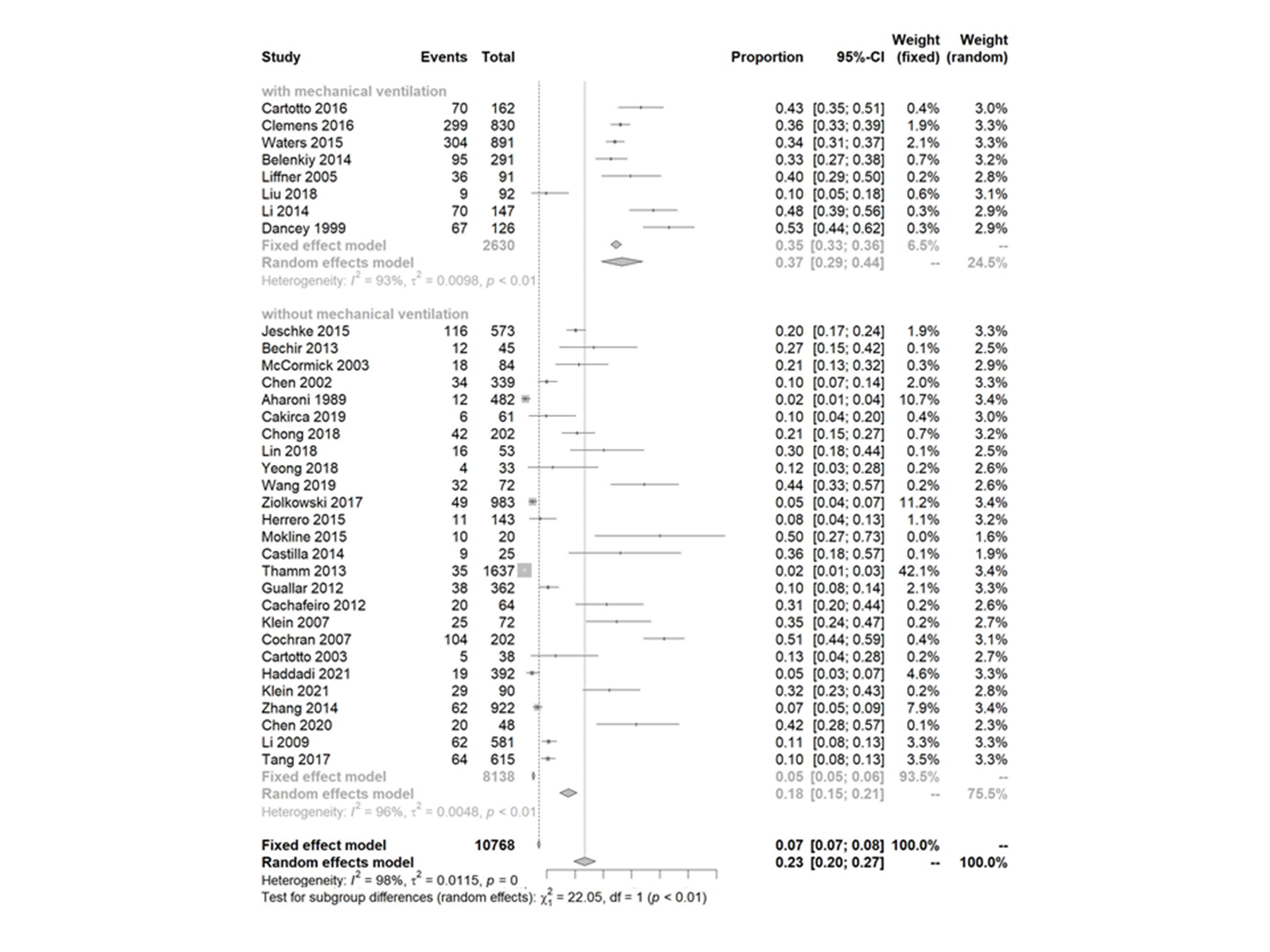

Supplement: Supplementary Figure 2 — Forest plot showing the incidence of acute respiratory distress syndrome in patients with burns and on mechanical ventilation. [file Image_2.TIF]

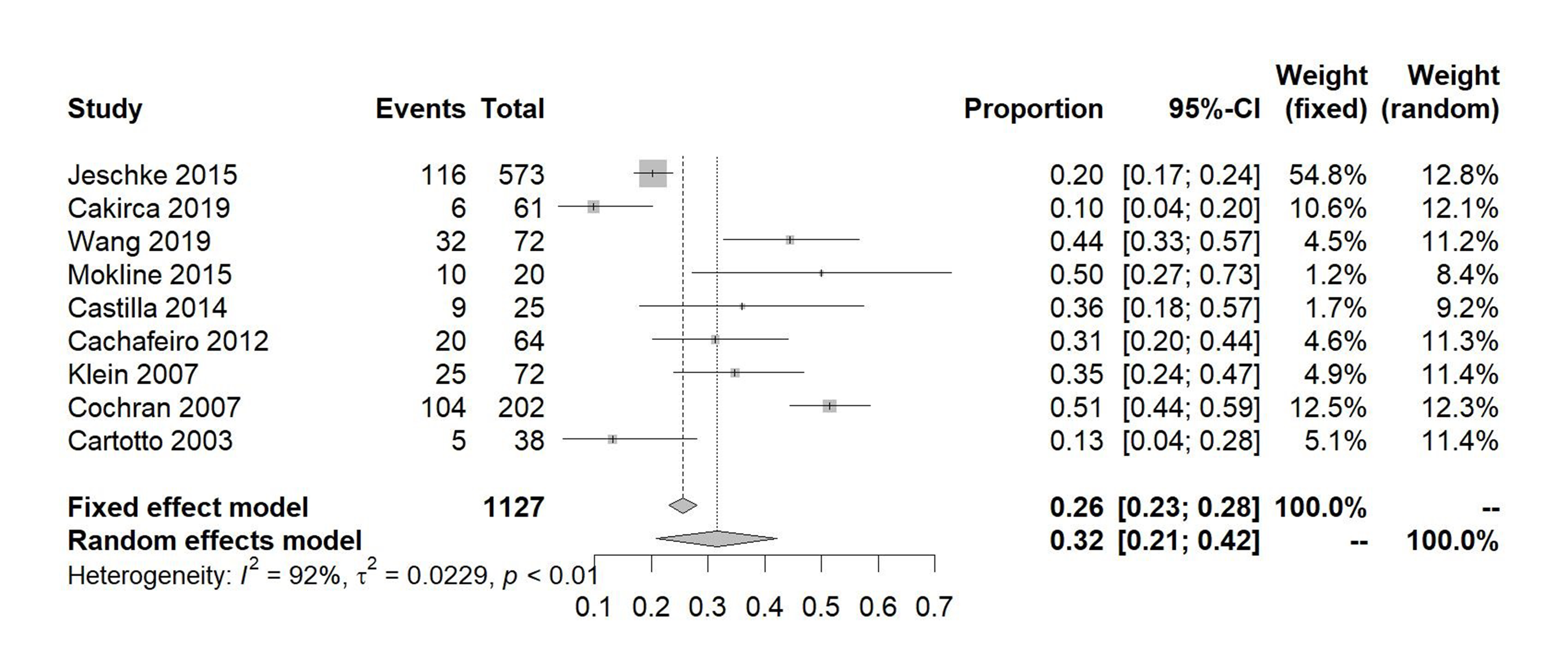

Supplement: Supplementary Figure 3 — Forest plot showing the incidence of acute respiratory distress syndrome in patients with burn and TBSA ≥ 20%. [file Image_3.tif]

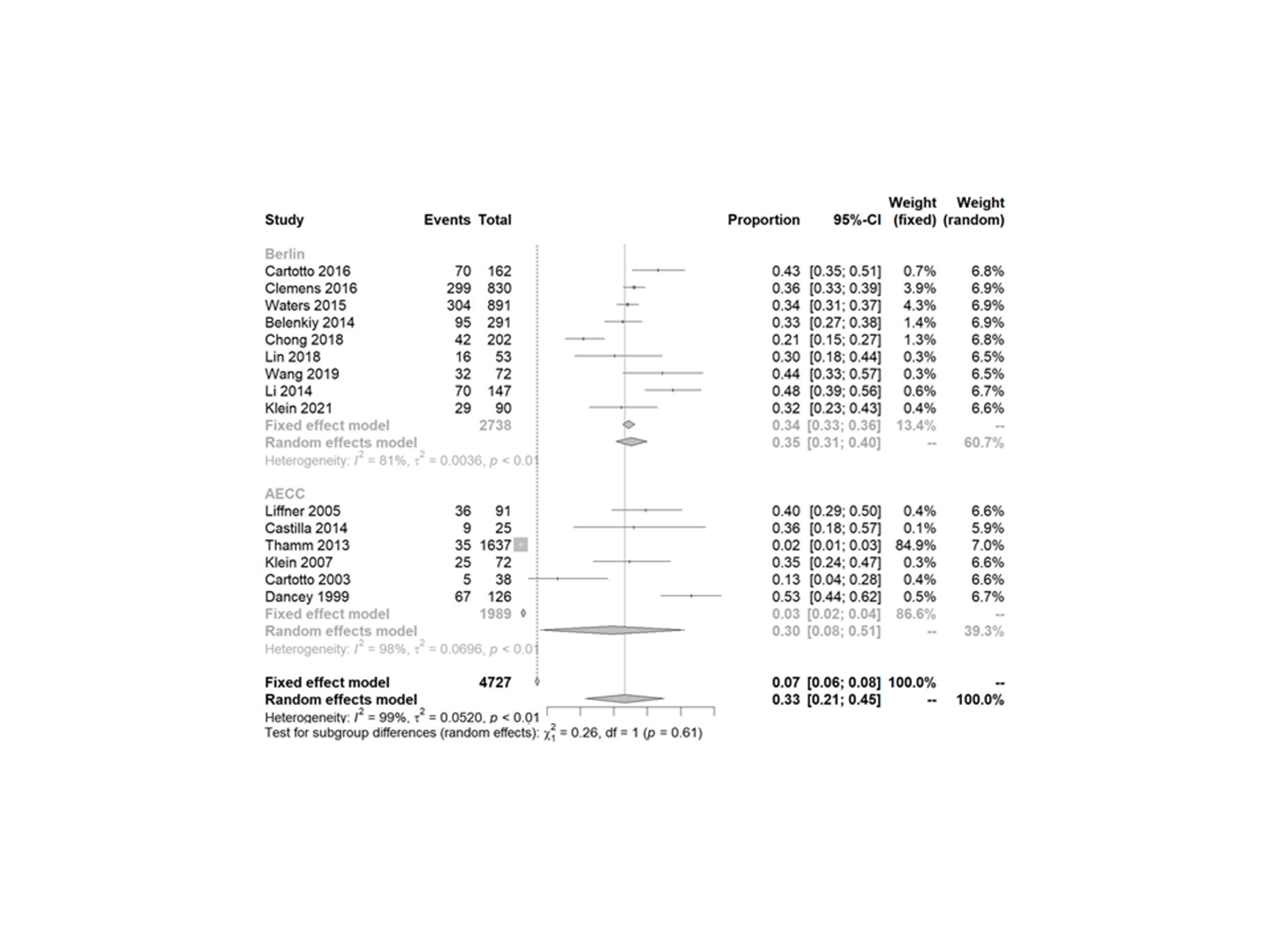

Supplement: Supplementary Figure 4 — Forest plot: incidence for the subgroups of American-European Consensus Conference definition and Berlin definition. [file Image_4.TIF]

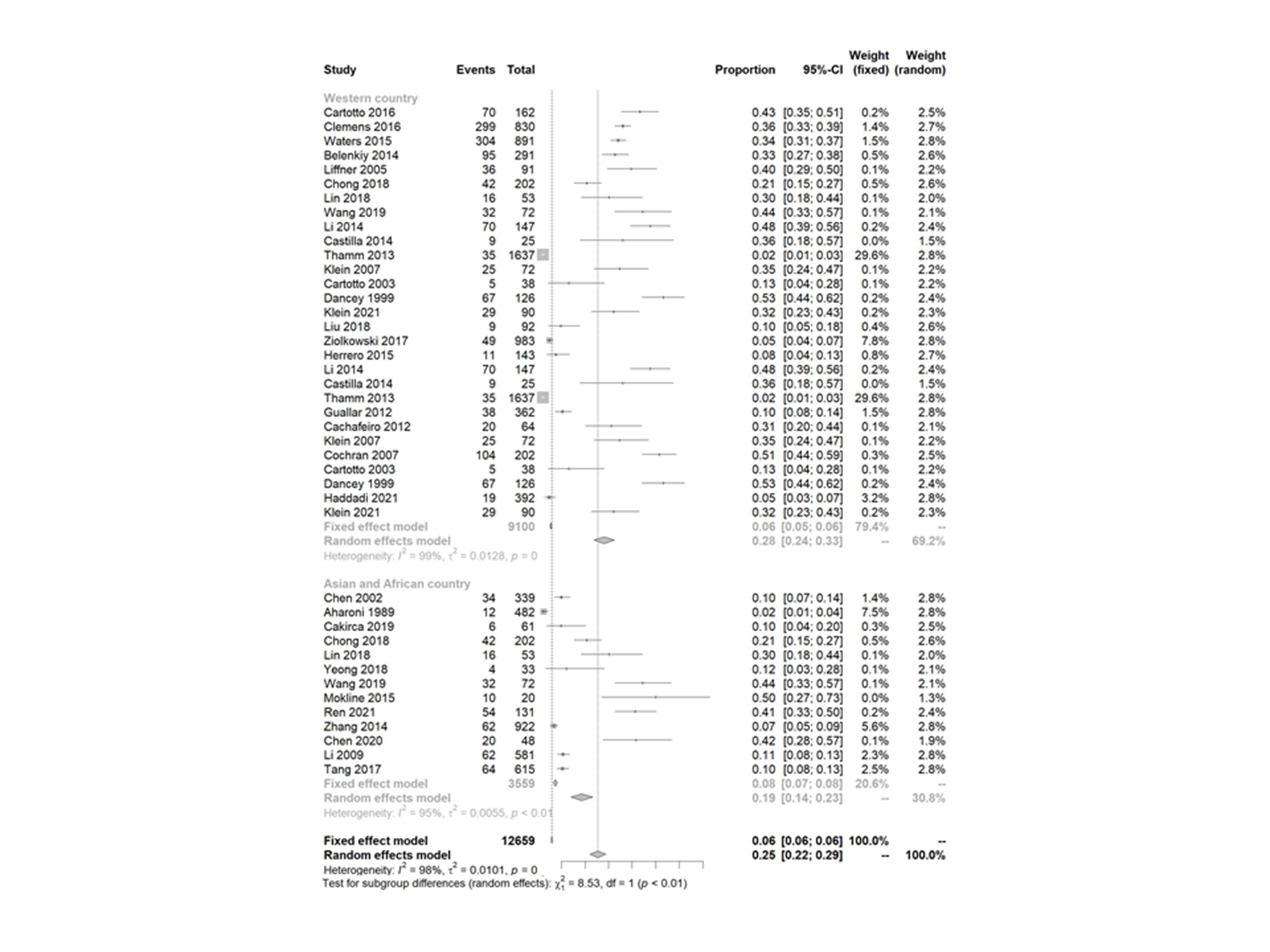

Supplement: Supplementary Figure 5 — Forest plot: incidence for the subgroups of Western countries and Asian and African countries. [file Image_5.TIF]

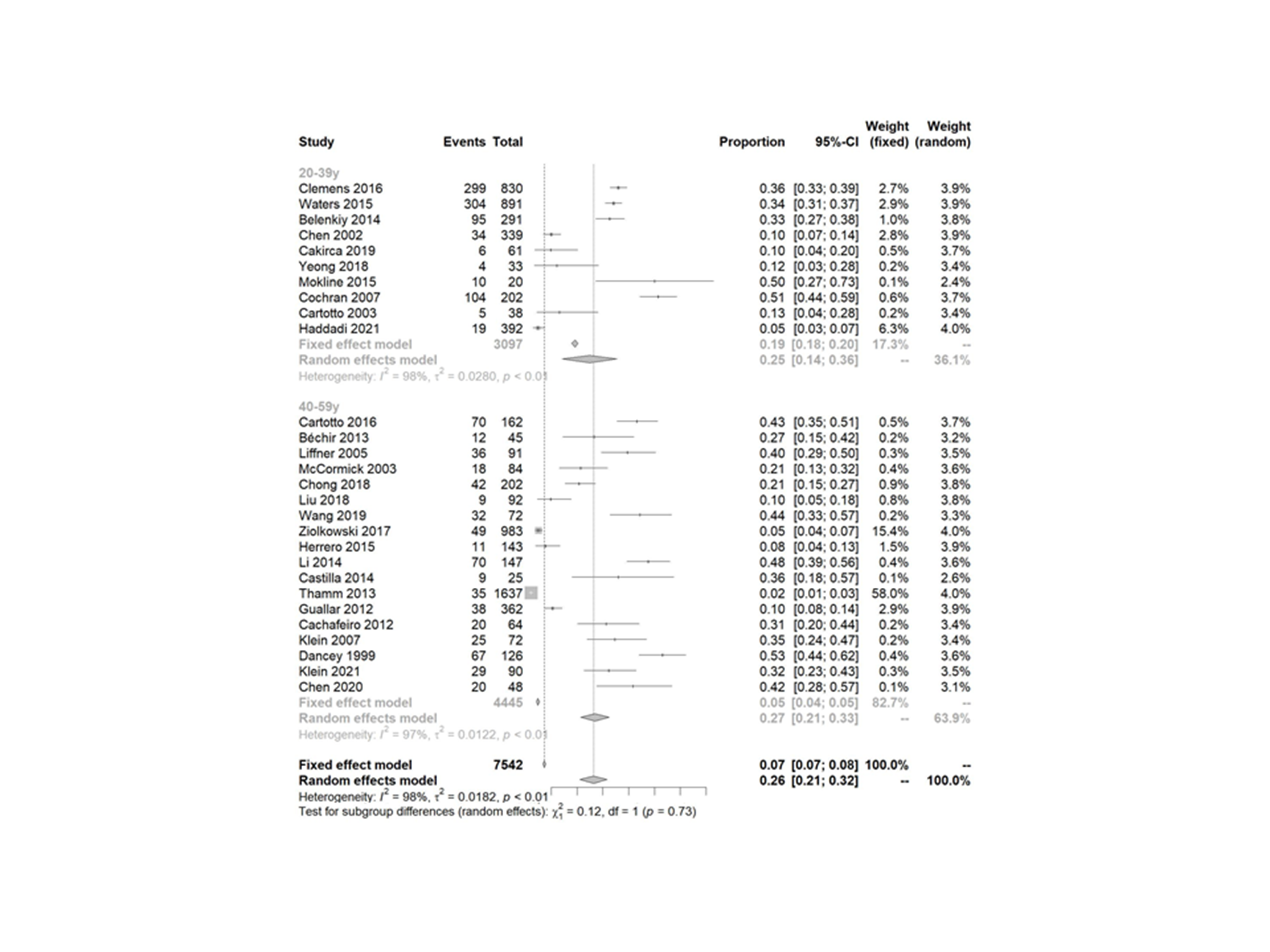

Supplement: Supplementary Figure 6 — Forest plot: incidence for the subgroups of 20–39- and 40−59-year-olds. [file Image_6.TIF]

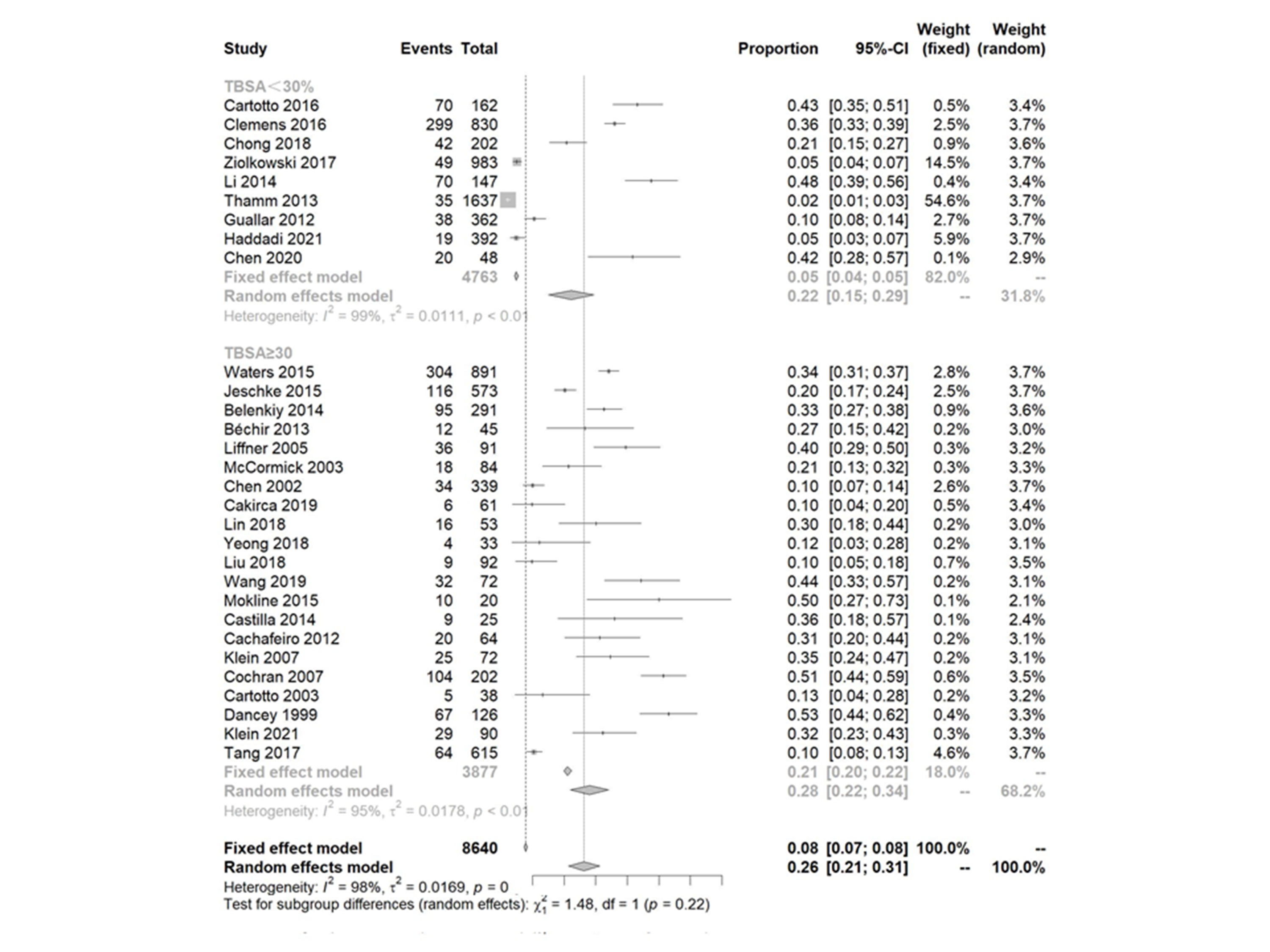

Supplement: Supplementary Figure 7 — Forest plot: incidence for the subgroups of mean total body surface area <30 and ≥30%. [file Image_7.TIF]

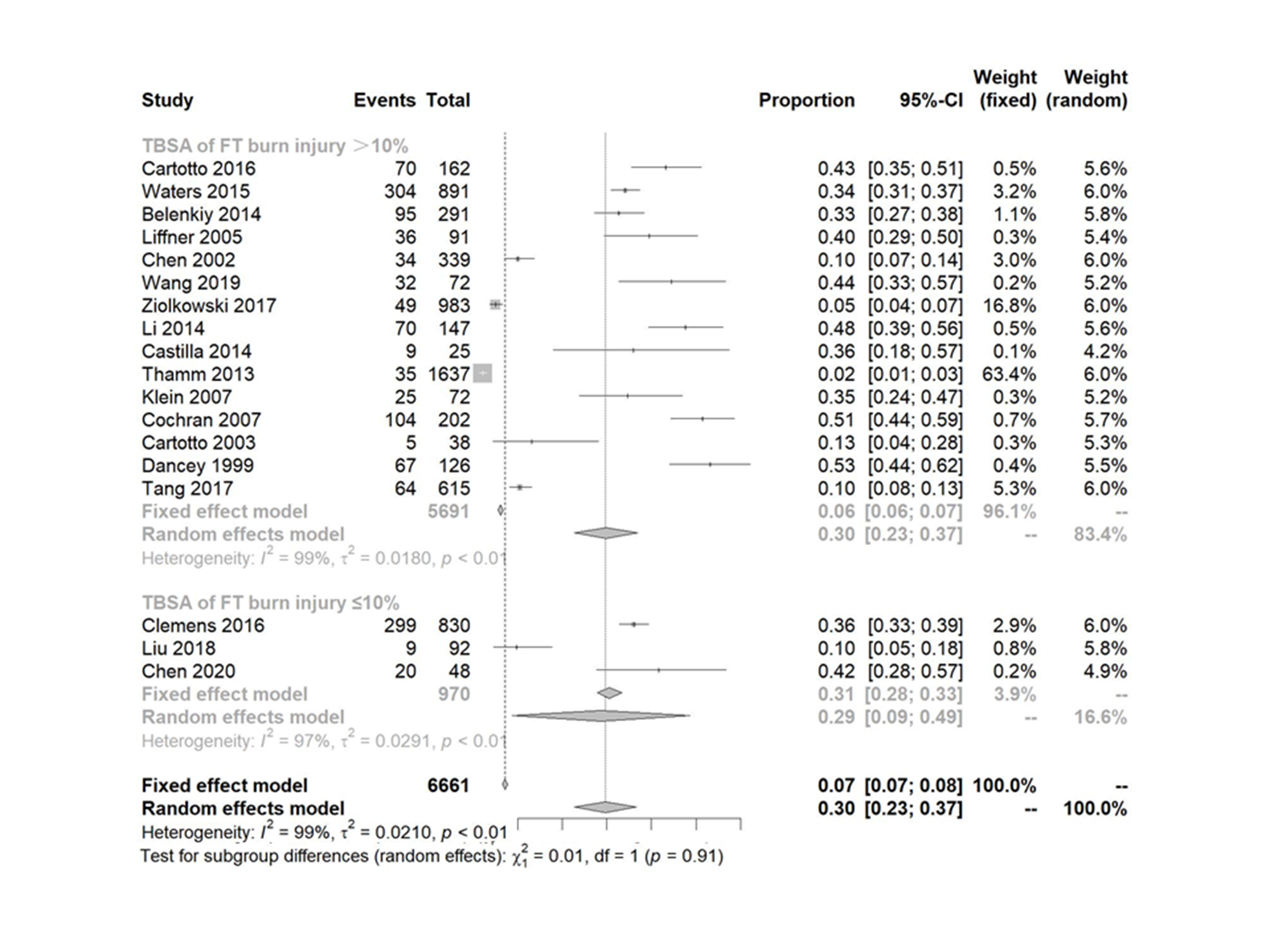

Supplement: Supplementary Figure 8 — Forest plot: incidence for the subgroups of inclusion of full-thickness burn injury ≤ 10 and >10%. [file Image_8.TIF]

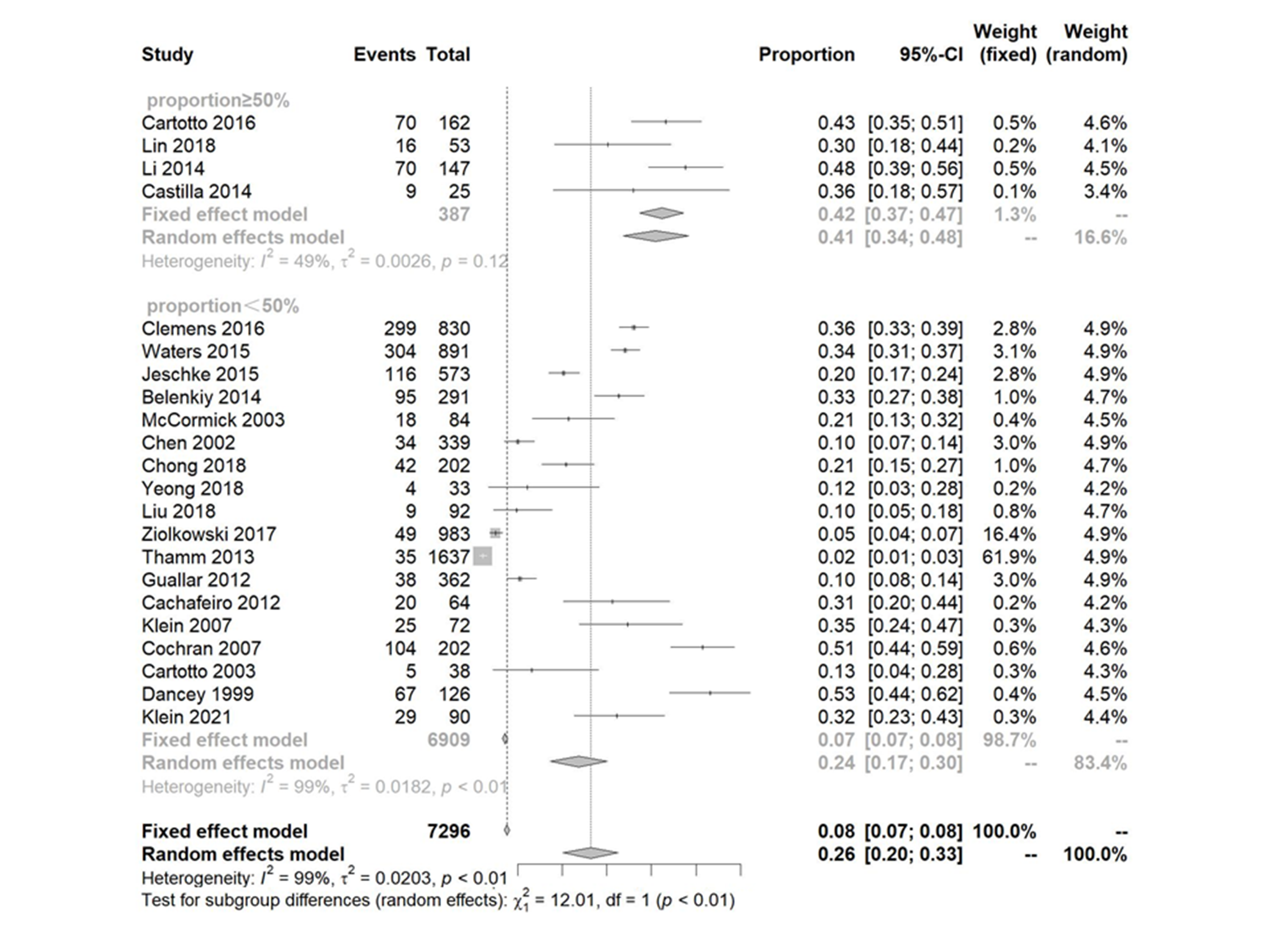

Supplement: Supplementary Figure 9 — Forest plot: incidence for the subgroups of patients with combined inhalation injury <50 and ≥50%. [file Image_9.TIF]

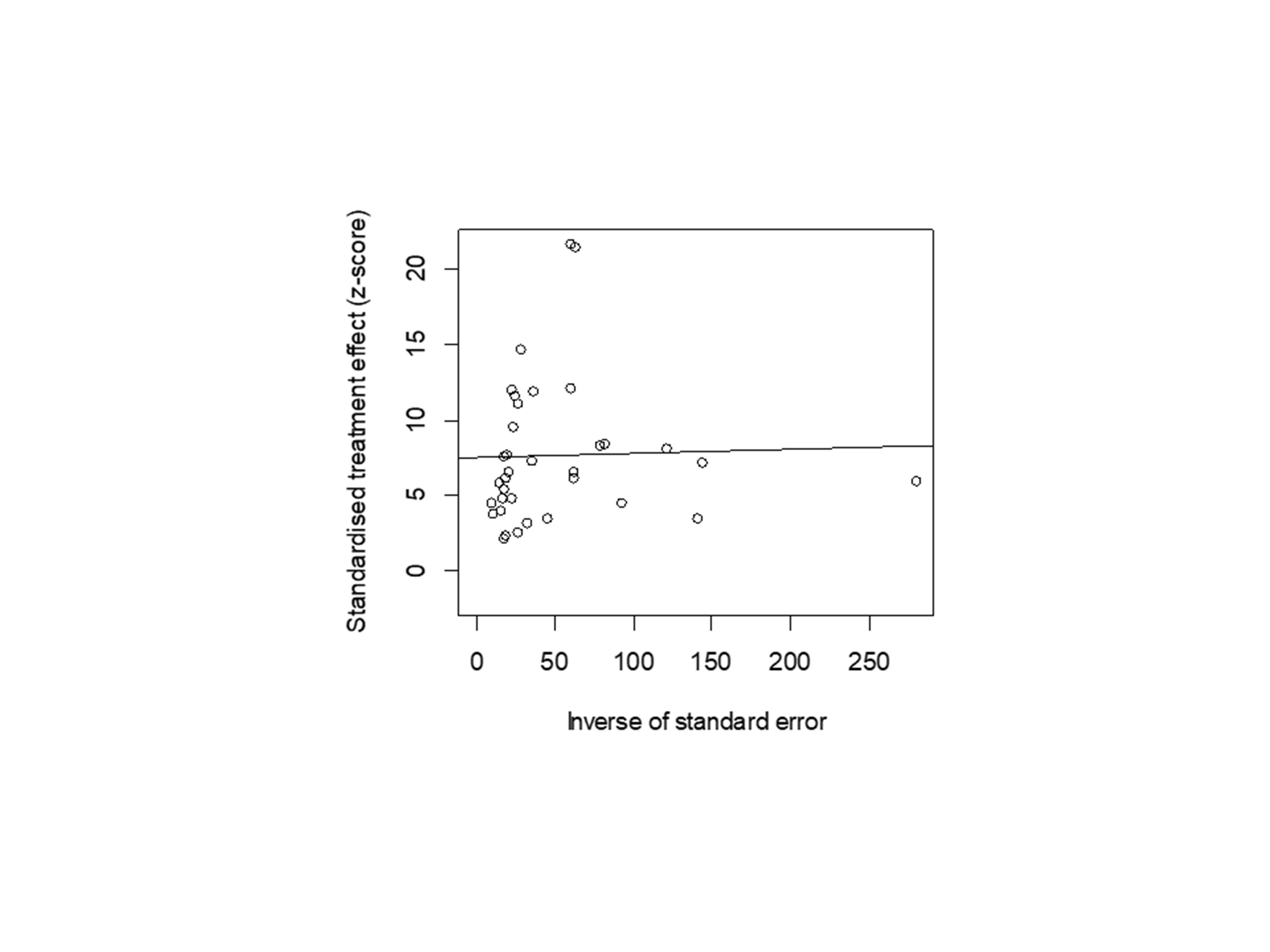

Supplement: Supplementary Figure 10 — Funnel plot of incidence studies. [file Image_10.TIF]

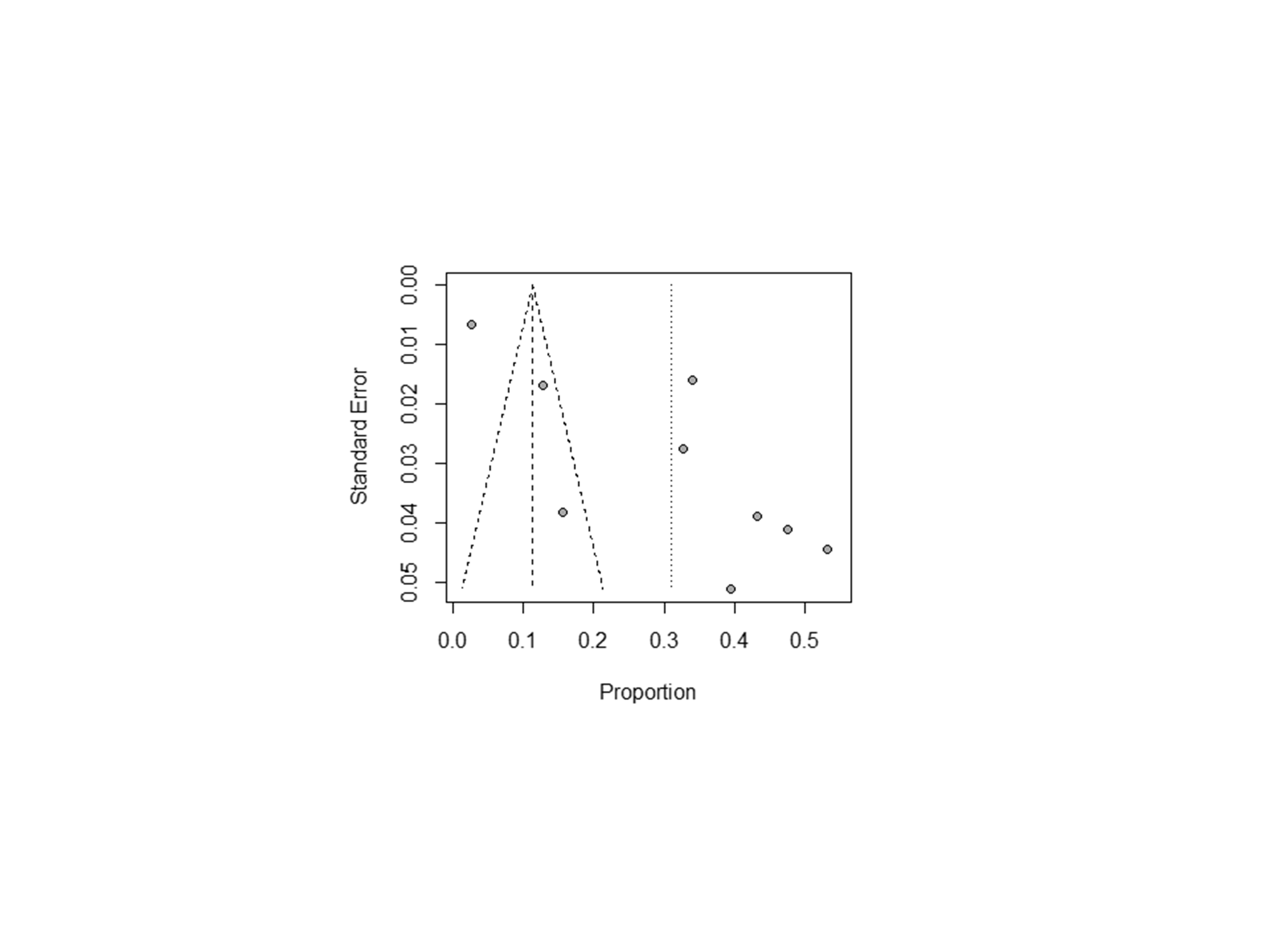

Supplement: Supplementary Figure 11 — Funnel plot of mortality studies. [file Image_11.TIF]

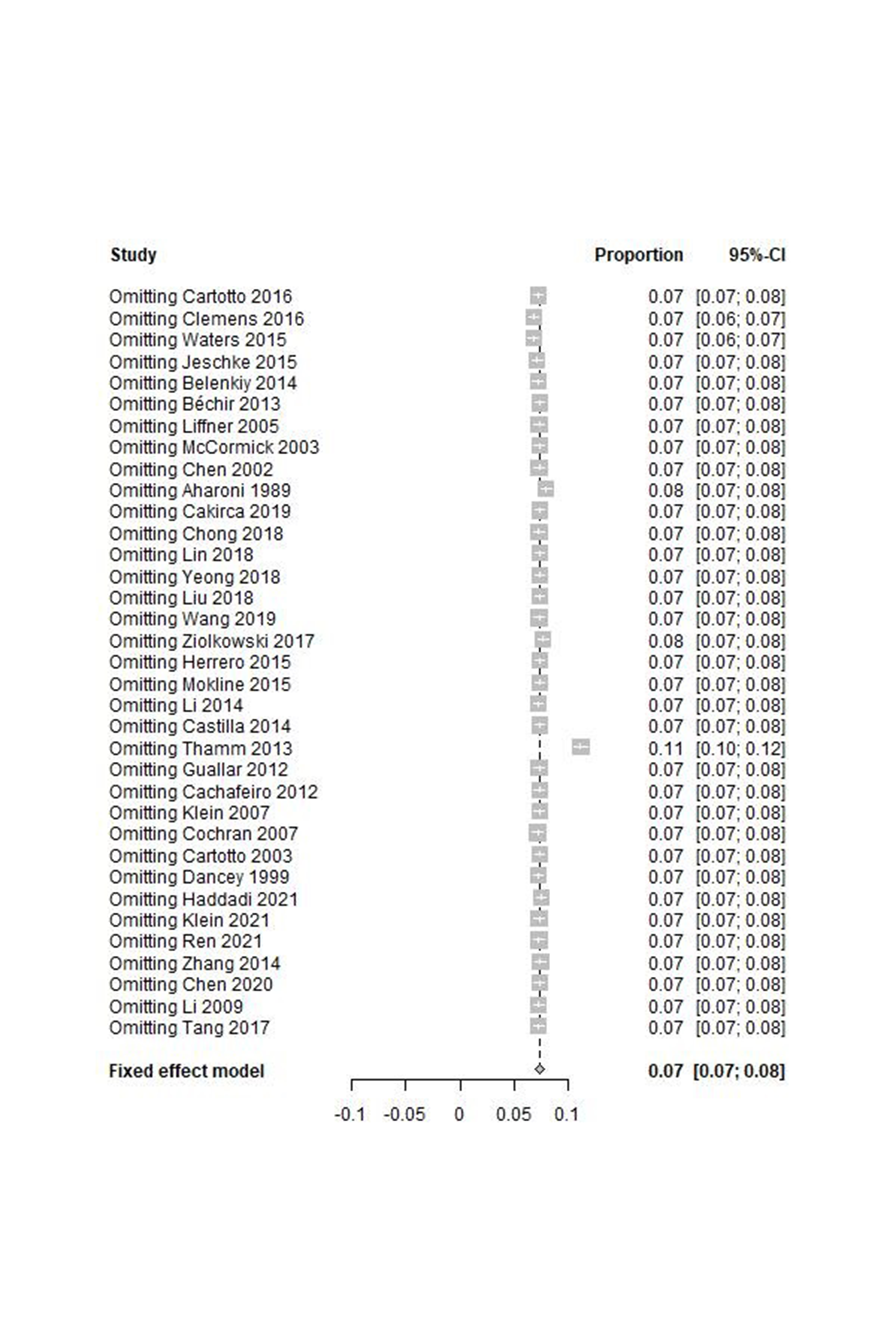

Supplement: Supplementary Figure 12 — Sensitive analysis for incidence of acute respiratory distress syndrome in patients with burns. [file Image_12.tif]

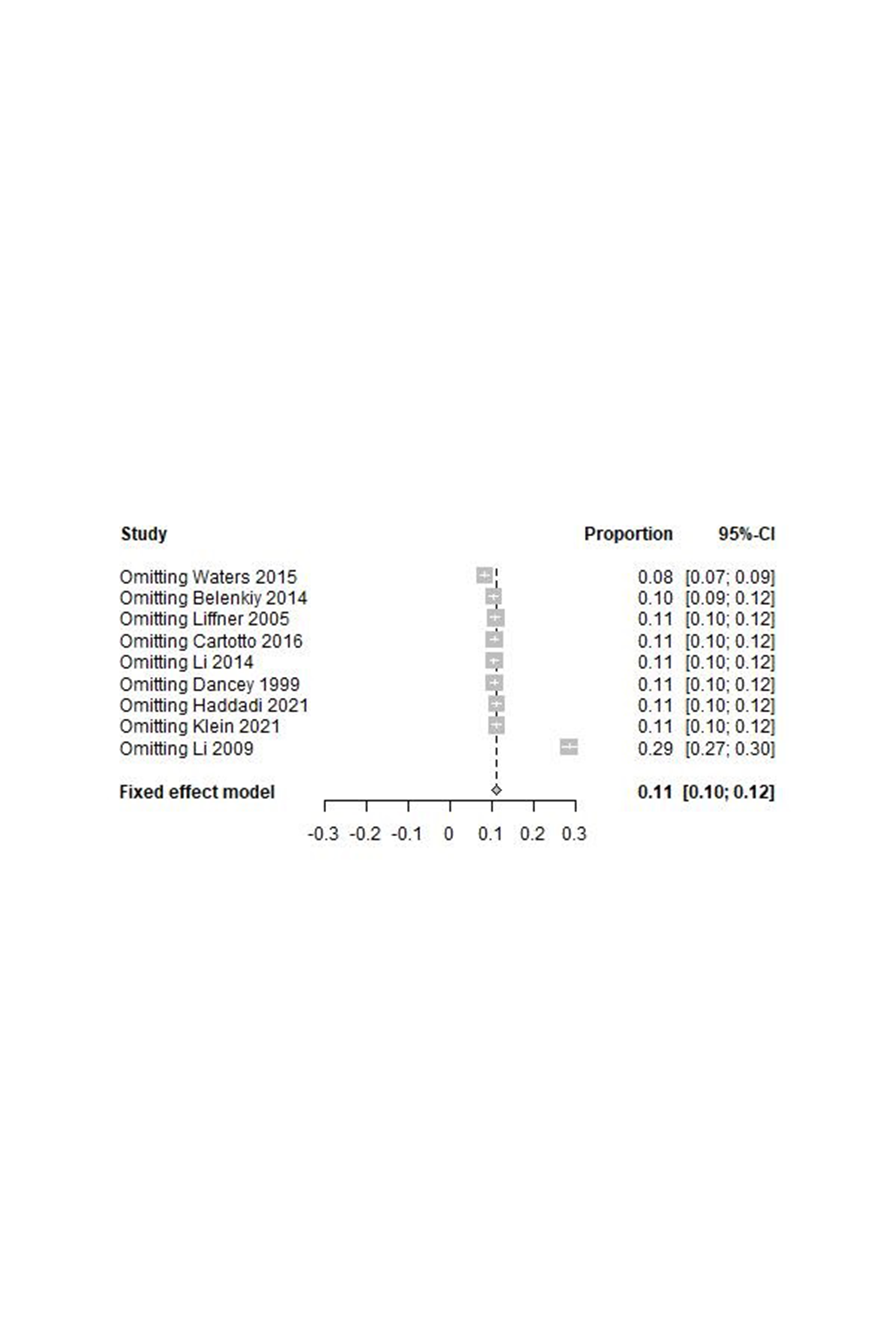

Supplement: Supplementary Figure 13 — Sensitive analysis for the mortality of acute respiratory distress syndrome in patients with burns. [file Image_13.tif]
