# Supplementary material for: Incidence and Mortality of Acute Respiratory Distress Syndrome in Patients With Burns: A Systematic Review and Meta-Analysis
Source: Front Med (Lausanne). 2021 Nov 15;8:709642. doi: 10.3389/fmed.2021.709642 (PMC8634659; doi:10.3389/fmed.2021.709642)
Supplement: Supplementary Table 1 — Characteristic of studies included in meta-analysis of incidence in burn patients. [file Table_1.docx]

**Supplementary Table 1. Characteristic of studies included in meta-analysis of incidence in burn patients**

| ID | | | **country, centers** | **event^a^** | | | **n^b^** | | **study style** | **Inclusion** | | | | **definition^d^** | | **mean age(y)** | **TBSA^e^**  **(%)** | | **quality score^f^** | | **FT^g^**  **(%)** | **II^h^**  **(%)** |  |  |  |
| --- | --- | --- | --- | --- | --- | --- | --- | --- | --- | --- | --- | --- | --- | --- | --- | --- | --- | --- | --- | --- | --- | --- | --- | --- | --- |
|  |  |  |  |  |  |  |  |  |  | **mechanical ventilation ^c^** | | **minimal TBSA (%)** | |  |  |  |  |  |  |  |  |  |  |  |  |
| Cartotto 2016 (5)^i^ | | Canada,1 | | | 70 | 162 | | - | | | with | | - | | Berlin | 48 | | 28 | | 85 | 13.0 | 62.0 |  |  |  |
| Clemens 2016 (6) | | USA，1 | | | 299 | 830 | | - | | | with | | - | | Berlin | 39.4 | | 27.6 | | 77 | 10.3 | 38.1 |  |  |  |
| Waters 2015 (7) | | USA,1 | | | 304 | 891 | | retrospective | | | with | | - | | Berlin | 39.5 | | 30 | | 69 | 17.3 | 38.3 |  |  |  |
| Jeschke 2015 (8) | | USA，6 | | | 116 | 573 | | - | | | without | | 20 | | - | - | | 48 | | 85 | - | 41.0 |  |  |  |
| Belenkiy 2014 (9) | | USA,1 | | | 95 | 291 | | - | | | with | | - | | Berlin | 26.3 | | 33.7 | | 88 | 25.5 | 44.0 |  |  |  |
| Béchir 2013 (10) | | Switzerland,1 | | | 12 | 45 | | - | | | without | | - | | - | 48 | | 31.5 | | 81 | - | - |  |  |  |
| Liffner 2005 (11) | | Sweden,1 | | | 36 | 91 | | - | | | with | | - | | AECC | 44.7 | | 31 | | 77 | 22.3 | - |  |  |  |
| McCormick 2003 (12) | | USA,1 | | | 18 | 84 | | - | | | without | | - | | - | 49.5 | | 34.1 | | 65 | - | 42.9 |  |  |  |
| Chen 2002 (13) | | China,1 | | | 34 | 339 | | retrospective | | | without | | - | | - | 36.7 | | 40.9 | | 50 | 0.2 | 19.0 |  |  |  |
| Aharoni 1989 (14) | | Israel,1 | | | 12 | 482 | | - | | | without | | - | | - | - | | - | | 65 | - | - |  |  |  |
| Çakırca 2019 (15) | | Ankara-Turkey,1 | | | 6 | 61 | | - | | | without | | 30 | | - | 36.5 | | 51 | | 77 | - | - |  |  |  |
| Chong 2018 (16) | | Singapore,1 | | | 42 | 202 | | retrospective | | | without | | - | | Berlin | 43 | | 12.1 | | 81 | - | 17.3 |  |  |  |
| Lin 2018 (17) | | China,1 | | | 16 | 53 | | - | | | without | | - | | Berlin | - | | 42.2 | | 81 | - | 56.6 |  |  |  |
| Yeong 2018 (18) | | China,1 | | | 4 | 33 | | - | | | without | | - | | - | 22 | | 43.2 | | 65 | - | 36.0 |  |  |  |
| Liu 2018 (19) | | Canada,1 | | | 9 | 92 | | - | | | with | | - | | - | 51 | | 35 | | - | 7.0 | 45.0 |  |  |  |
| Wang 2019 (20) | | China,1 | | | 32 | 72 | | - | | | without | | 50 | | Berlin | 40.3 | | 69.3 | | 88 | 26.1 | - |  |  |  |
| Ziolkowski 2017(21) | | Canada,1 | | | 49 | 983 | | - | | | without | | - | | - | 48 | | 14 | | 85 | 49.0 | 14.0 |  |  |  |
| Herrero 2015 (22) | | Spain,1 | | | 11 | 143 | | prospective | | | without | | - | | - | 46.98 | | 22.82 | | - | - | - |  |  |  |
| Mokline 2015 (23) | | Tunisia,1 | | | 10 | 20 | | - | | | without | | 20 | | - | 36 | | 44 | | - | - | - |  |  |  |
| Li 2014 (24) | | Canada,1 | | | 70 | 147 | | retrospective | | | with | | - | | Berlin | 47 | | 28 | | - | 12.0 | 67.0 |  |  |  |
| Castilla 2014 (25) | | Spain,1 | | | 9 | 25 | | - | | | without | | 20 | | AECC | 42 | | 33 | | 88 | 40.0 | 72.0 |  |  |  |
| Thamm 2013 (26) | | Germany,1 | | | 35 | 1637 | | retrospective | | | without | | - | | AECC | 41 | | 21 | | 77 | 37.9 | 36.5 |  |  |  |
| Guallar 2012 (27) | | Spain,1 | | | 38 | 362 | | retrospective | | | without | | - | | - | 46.96 | | 20.95 | | - | - | 23.2 |  |  |  |
| Cachafeiro 2012 (28) | | Spain,1 | | | 20 | 64 | | prospective | | | without | | 20 | | - | 48 | | 40 | | - | - | 35.9 |  |  |  |
| Klein 2007 (29) | | USA,5 | | | 25 | 72 | | - | | | without | | 20 | | AECC | 40.6 | | 44.5 | | 69 | 30.7 | 41.7 |  |  |  |
| Cochran 2007 (30) | | USA,1 | | | 104 | 202 | | retrospective | | | without | | 20 | | - | 36.9 | | 41.1 | | 69 | 18.6 | 34.7 |  |  |  |
| Cartotto 2003 (31) | | Canada,1 | | | 5 | 38 | | prospective | | | without | | 20 | | AECC | 39 | | 36 | | 69 | 16.7 | 30.0 |  |  |  |
| Dancey 1999 (32) | | Canada,1 | | | 67 | 126 | | retrospective | | | with | | - | | AECC | 46.9 | | 37.3 | | 85 | 61.9 | 34.9 |  |  |  |
| Haddadi 2021 (33) | | North Iran,1 | | | 19 | 392 | | retrospective | | | without | | - | | - | 37.14 | | 29.31 | | 88 | - | - |  |  |  |
| Klein 2021 (34) | | Switzerland,1 | | | 29 | 90 | | retrospective | | | without | | 15 | | Berlin | 52 | | 31.5 | | 88 | - | 27 |  |  |  |
| Ren 2021 (35) | | China,1 | | | 54 | 131 | | retrospective | | |  | | 10 | | - | - | | - | | 69 | - | - |  |  |  |
| Zhang 2014 (36) | | China,1 | | | 62 | 922 | | retrospective | | | without | | 10 | | - | - | | - | | 65 | - | - |  |  |  |
| Chen 2020 (37) | | China,1 | | | 20 | 48 | | retrospective | | | without | | 10 | | - | 51.05 | | 22.13 | | 88 | 7.41 | - |  |  |  |
| Li 2009 (38) | | China,1 | | | 62 | 581 | | retrospective | | | without | | - | | - |  | |  | |  |  |  |  |  |  |
| Tang 2017 (39) | | China,1 | | | 64 | 615 | | retrospective | | | without | | 10 | | - | - | | 48.9% | | 88 | 19.8 | - |  |  |  |
|  | (a) event: occur acute respiratory distress syndrome  (b) n: total sample size  (c) inclusion criteria of patients with mechanical ventilation or without  (d) definition: which definition of acute respiratory distress syndrome used in the studies, the American-European Consensus Conference (AECC) definition or the Berlin definition  (e) TBSA: total body surface area  (f) quality score: the score of methodological quality of the study  (g) FT: full-thickness (FT) burn injury  (h) II: the proportion of patients combined inhalation injury of the total sample  (i) Numbers in parentheses refer to the reference numbers in the main manuscript  (j) “-” means the date is not available | | | | | | | | | | | | | | | | | | | | | |  |  | without |
