# Supplementary material for: Incidence and Mortality of Acute Respiratory Distress Syndrome in Patients With Burns: A Systematic Review and Meta-Analysis
Source: Front Med (Lausanne). 2021 Nov 15;8:709642. doi: 10.3389/fmed.2021.709642 (PMC8634659; doi:10.3389/fmed.2021.709642)
Supplement: Supplementary Table 2 — Characteristic of studies included in meta-analysis of mortality in burn patients. [file Table_2.docx]

**Supplementary Table 2. Characteristic of studies included in meta-analysis of mortality in burn patients**

| ID | | | **country, centers** | **event^a^** | | | **n^b^** | | **study style** | **Inclusion** | | | | **definition^d^** | | **mean age(y)** | **TBSA^e^**  **(%)** | | **quality score^f^** | | **FT^g^**  **(%)** | **II^h^**  **(%)** |  |  |  |
| --- | --- | --- | --- | --- | --- | --- | --- | --- | --- | --- | --- | --- | --- | --- | --- | --- | --- | --- | --- | --- | --- | --- | --- | --- | --- |
|  |  |  |  |  |  |  |  |  |  | **mechanical ventilation ^c^** | | **minimal TBSA (%)** | |  |  |  |  |  |  |  |  |  |  |  |  |
| Waters 2015 (7) ^i^ | | USA,1 | | | 304 | 891 | | retrospective | | | with | | - | | Berlin | 39.5 | | 30 | | 69 | 17.3 | 38.3 |  |  |  |
| Belenkiy 2014 (9) | | USA,1 | | | 95 | 291 | | - | | | with | | - | | Berlin | 26.3 | | 33.7 | | 88 | 25.5 | 44.0 |  |  |  |
| Liffner 2005 (11) | | Sweden,1 | | | 36 | 91 | | - | | | with | | - | | AECC | 44.7 | | 31 | | 77 | 22.3 | - |  |  |  |
| Cartotto 2016 (5) | | Canada,1 | | | 70 | 162 | | - | | | with | | - | | Berlin | 48 | | 28 | | 85 | 13.0 | 62.0 |  |  |  |
| Li 2014 (24) | | Canada,1 | | | 70 | 147 | | retrospective | | | with | | - | | Berlin | 47 | | 28 | | - | 12.0 | 67.0 |  |  |  |
| Dancey 1999 (32) | | Canada,1 | | | 67 | 126 | | retrospective | | | with | | - | | AECC | 46.9 | | 37.3 | | 85 | 61.9 | 34.9 |  |  |  |
| Haddadi 2021 (33) | | North Iran，1 | | | 50 | 392 | | retrospective | | | without | | - | | - | 37.14 | | 29.31 | | 88 | - | - |  |  |  |
| Klein 2021 (34) | | Switzerland,1 | | | 14 | 90 | | retrospective | | | without | | 15 | | Berlin | 52 | | 31.5 | | 88 | - | -27 |  |  |  |
| Li 2009 (38) | | China,1 | | | 15 | 581 | | retrospective | | | without | | - | | - | - | | - | | 65 | - | - |  |  |  |
|  | (a) event: acute respiratory distress syndrome related death of this study  (b) total acute respiratory distress syndrome patient in this study  (c) inclusion criteria of patients with mechanical ventilation or without  (d) definition: which definition of acute respiratory distress syndrome used in the studies, the American-European Consensus Conference (AECC) definition or the Berlin definition  (e) TBSA: total body surface area  (f) quality score: the score of methodological quality of the study  (g) FT: full-thickness (FT) burn injury  (h) II: the proportion of patients combined inhalation injury of the total sample  (i) Numbers in parentheses refer to the reference numbers in the main manuscript  (j) “-” means the date is not available | | | | | | | | | | | | | | | | | | | | | |  |  | without |
