# Supplementary material for: Incidence and Mortality of Acute Respiratory Distress Syndrome in Patients With Burns: A Systematic Review and Meta-Analysis
Source: Front Med (Lausanne). 2021 Nov 15;8:709642. doi: 10.3389/fmed.2021.709642 (PMC8634659; doi:10.3389/fmed.2021.709642)
Supplement: Supplementary Table 3 — Quality assessment of the included studies on incidence of acute respiratory distress syndrome in burn patient with full-text. [file Table_3.docx]

**Supplementary Table 3. Quality assessment of the included studies on incidence of acute respiratory distress syndrome in burn patient with full-text**

| **ID** | **1** | **2a** | **2b** | **3a** | **3b** | **4** | **5a** | **5b** | **6** | **7** | **8** | **9** | **10** | **11** | **12** | **13** | **Total score** | **Quality score (%)** |
| --- | --- | --- | --- | --- | --- | --- | --- | --- | --- | --- | --- | --- | --- | --- | --- | --- | --- | --- |
| Cartotto 2016 | 1 | 0.5 | 0.5 | 0.5 | 0 | 1 | 0.5 | 0 | 1 | 1 | 1 | 1 | 1 | 1 | 0 | 1 | 11 | 85 |
| Clemens 2016 | 1 | 0.5 | 0.5 | 0.5 | 0 | 1 | 0.5 | 0 | 1 | 1 | 1 | 1 | 1 | 1 | 0 | 0 | 10 | 77 |
| Waters 2015 | 1 | 0.5 | 0.5 | 0.5 | 0 | 1 | 0 | 0 | 0.5 | 1 | 1 | 1 | 1 | 1 | 0 | 0 | 9 | 69 |
| Jeschke 2015 | 1 | 0.5 | 0.5 | 0 | 0.5 | 1 | 0 | 0 | 0.5 | 1 | 1 | 1 | 1 | 1 | 1 | 1 | 11 | 85 |
| Belenkiy 2014 | 1 | 0.5 | 0.5 | 0.5 | 0 | 1 | 0.5 | 0 | 1 | 1 | 1 | 1 | 1 | 1 | 0 | 1 | 11 | 85 |
| Béchir 2013 | 0.5 | 0.5 | 0.5 | 0.5 | 0.5 | 1 | 0 | 0 | 1 | 1 | 1 | 1 | 1 | 1 | 0 | 1 | 10.5 | 81 |
| Liffner 2005 | 1 | 0.5 | 0.5 | 0.5 | 0.5 | 1 | 0.5 | 0 | 0.5 | 1 | 1 | 1 | 1 | 1 | 0 | 0 | 10 | 77 |
| McCormick 2003 | 1 | 0 | 0.5 | 0.5 | 0 | 1 | 0 | 0 | 1 | 1 | 1 | 0.5 | 1 | 1 | 0 | 0 | 8.5 | 65 |
| Chen 2002 | 1 | 0.5 | 0.5 | 0 | 0 | 1 | 0 | 0 | 0.5 | 1 | 0 | 1 | 1 | 0 | 0 | 0 | 6.5 | 50 |
| Aharoni 1989 | 0.5 | 0.5 | 0.5 | 0.5 | 0 | 1 | 0.5 | 0 | 1 | 1 | 0 | 1 | 1 | 1 | 0 | 0 | 8.5 | 65 |
| Çakırca 2019 | 1 | 0.5 | 0.5 | 0.5 | 0 | 1 | 0 | 0 | 0.5 | 1 | 1 | 1 | 1 | 1 | 1 | 0 | 10 | 77 |
| Chong 2018 | 1 | 0.5 | 0.5 | 0 | 0.5 | 0.5 | 0 | 0 | 0.5 | 1 | 1 | 1 | 1 | 1 | 1 | 1 | 10.5 | 81 |
| Lin 2018 | 1 | 0.5 | 0.5 | 0 | 0 | 1 | 0.5 | 0 | 1 | 1 | 1 | 1 | 1 | 1 | 0 | 1 | 10.5 | 81 |
| Wang 2019 | 1 | 0.5 | 0.5 | 0.5 | 0.5 | 1 | 0 | 0 | 0.5 | 1 | 1 | 1 | 1 | 1 | 1 | 1 | 11.5 | 88 |
| Ziolkowski 2017 | 1 | 0.5 | 0.5 | 0.5 | 0 | 1 | 0 | 0 | 0.5 | 1 | 1 | 1 | 1 | 1 | 1 | 1 | 11 | 85 |
| Castilla 2014 | 1 | 0.5 | 0.5 | 0 | 0.5 | 1 | 0 | 0 | 1 | 1 | 1 | 1 | 1 | 1 | 1 | 1 | 11.5 | 88 |
| Thamm 2013 | 1 | 0 | 0.5 | 0 | 0 | 1 | 0 | 0 | 0.5 | 1 | 1 | 1 | 1 | 1 | 1 | 1 | 10 | 77 |
| Klein 2007 | 1 | 0.5 | 0.5 | 0 | 0 | 1 | 0 | 0.5 | 1 | 1 | 1 | 0.5 | 1 | 1 | 0 | 0 | 9 | 69 |
| Cochran 2007 | 1 | 0.5 | 0.5 | 0.5 | 0 | 1 | 0 | 0 | 1 | 1 | 1 | 0.5 | 1 | 1 | 0 | 0 | 9 | 69 |
| Cartotto 2003 | 1 | 0.5 | 0.5 | 0.5 | 0 | 1 | 0.5 | 0.5 | 0.5 | 1 | 0 | 1 | 1 | 1 | 0 | 0 | 9 | 69 |
| Dancey 1999 | 1 | 0.5 | 0.5 | 0 | 0 | 1 | 0.5 | 0.5 | 1 | 1 | 1 | 1 | 1 | 1 | 1 | 0 | 11 | 85 |
| Haddadi 2021 | 1 | 0.5 | 0.5 | 0 | 0 | 1 | 0.5 | 0 | 1 | 1 | 1 | 1 | 1 | 1 | 1 | 1 | 11.5 | 88 |
| Klein 2021 | 1 | 0.5 | 0.5 | 0.5 | 0 | 1 | 0 | 0.5 | 0.5 | 1 | 1 | 1 | 1 | 1 | 1 | 1 | 11.5 | 88 |
| Ren 2021 | 1 | 0 | 0.5 | 0 | 0.5 | 0.5 | 0 | 0 | 0.5 | 1 | 0 | 1 | 1 | 1 | 1 | 1 | 9 | 69 |
| Zhang 2014 | 1 | 0.5 | 0.5 | 0 | 0 | 0.5 | 0 | 0.5 | 0.5 | 1 | 0 | 1 | 1 | 0 | 1 | 1 | 8.5 | 65 |
| Chen 2020 | 1 | 0.5 | 0.5 | 0.5 | 0.5 | 1 | 0 | 0 | 0.5 | 1 | 1 | 1 | 1 | 1 | 1 | 1 | 11.5 | 88 |
| Li 2009 | 1 | 0.5 | 0.5 | 0 | 0 | 1 | 1 | 0 | 0.5 | 1 | 0 | 1 | 1 | 1 | 0 | 0 | 8.5 | 65 |
| Tang 2017 | 1 | 0.5 | 0.5 | 0.5 | 0.5 | 1 | 0 | 0.5 | 0 | 1 | 1 | 1 | 1 | 1 | 1 | 1 | 11.5 | 88 |
| All included studies are assessed by 13 questions based on the Critical appraisal of the health research literature: prevalence or incidence of a health problem and the STROBE guidelines [5]. 1 = yes (high quality); and 0= no (low quality). The quality score is the sum of all answers, with the percentage of questions answered as yes. | | | | | | | | | | | | | | | | | | |
