# Supplementary material for: Incidence and Mortality of Acute Respiratory Distress Syndrome in Patients With Burns: A Systematic Review and Meta-Analysis
Source: Front Med (Lausanne). 2021 Nov 15;8:709642. doi: 10.3389/fmed.2021.709642 (PMC8634659; doi:10.3389/fmed.2021.709642)
Supplement: Appendix 1 — Search strategies of PubMed, Embase, CINAHL databases, and China National Knowledge Infrastructure database. [file Data_Sheet_1.doc]

**Supplementary** **Material**

**Incidence and Mortality of Acute Respiratory Distress Syndrome in Burn Patients: A Systematic Review and Meta-Analysis**

**Appendix 1. Search strategies of PubMed, Embase and CINAHL**

**PubMed search**

| ALI/ARDS: | Lung Injury[MeSH Terms] OR Acute Lung Injury[MeSH Terms] OR Ventilator-Induced Lung Injury[MeSH Terms] OR Respiratory Distress Syndrome, Adult[MeSH Terms] OR acute lung injury[Title/Abstract] OR acute respiratory distress[Title/Abstract] OR ARDS[Title/Abstract] OR ALI/ARDS[Title/Abstract] OR ARDS/ALI[Title/Abstract] |
| --- | --- |
| Incidence / Mortality: | incidence[Title/Abstract] OR prevalence[Title/Abstract] OR Mortality[MeSH Terms] OR Mortality[MeSH Subheading] OR Outcome Assessment AND (Health Care) AND MeSH Terms) OR mortality[Title/Abstract] OR outcome*[Title/Abstract] OR ventilator-free day*[Title/Abstract] OR length of hospital stay[Title/Abstract]) |
| burn | (burns[MeSH Terms] OR burn*[Title/Abstract] OR Burn Unit[MeSH Terms] OR Burn Unit[Title/Abstract] OR Unit, Burn[Title/Abstract] OR Units, Burn[Title/Abstract] OR Burn Center[Title/Abstract] OR Burn Centers[Title/Abstract] OR Centers, Burn[Title/Abstract] OR Center, Burn[Title/Abstract] OR thermal injury[Title/Abstract] |
| Filter: | English and humans"[Filter] AND "loattrfull text"[Filter] NOT "review"[Filter] |

**Embase search**：

| 1 'lung injury'/exp/mj (14,462) |
| --- |
| 2 'ventilator induced lung injury'/exp (1,947) |
| 3 'adult respiratory distress syndrome'/exp (32,853) |
| 4 'acute lung injury':ab,ti OR 'acute respiratory distress':ab,ti OR 'ards':ab,ti OR 'ali':ab,ti (43,529) |
| 5 1 OR 2 OR 3 OR 4 (68,327) |
| 6 'epidemiology'/exp/mj (381,311) |
| 7 'incidence'/exp/mj (23,099) |
| 8 'prevalence'/exp/mj (58,414) |
| 9 ' mortality'/exp/mj (138,932) |
| 10 'outcome assessment'/exp (466,036) |
| 11 'incidence':ab,ti OR 'prevalence':ab,ti OR 'mortality':ab,ti OR 'outcome*':ab,ti OR 'ventilator-free day*':ab,ti OR 'length of hospital stay':ab,ti (4,110,126) |
| 12 6 OR 7 OR 8 OR 9 OR 10 OR 11 (4,401,112) |
| 13 'burn'/exp/mj (46,797) |
| 14 'burn unit'/exp/mj (274) |
| 15 'burn*':ab,ti OR 'burn unit*':ab,ti OR 'unit*, burn':ab,ti OR 'burn center*':ab,ti OR 'centers*, burn':ab,ti OR 'thermal injury':ab,ti (122,132) |
| 16 13 OR 14 OR 15 OR 16 (129,991) |
| 17 5 AND 12 AND 16 (460) |
| 18 5 AND 12 AND 16 AND [humans]/lim AND [english]/lim (337) |

**The cochrane library search**：

| "Lung Injury" OR "Acute Lung Injury" OR "Ventilator-Induced Lung Injury" OR "Respiratory Distress Syndrome, Adult" OR "acute respiratory distress" OR "ARDS" OR "ALI" in Title Abstract Keyword AND "Epidemiology" OR "Incidence" OR "Prevalence" OR "incidence" OR "Mortality" OR "outcome*" OR "ventilator-free day*" OR "length of hospital stay" in Title Abstract Keyword AND "burn*" OR "thermal injury" in Title Abstract Keyword |
| --- |

CINAHL search：

| AB ( "Lung Injury" OR "Acute Lung Injury" OR "Ventilator-Induced Lung Injury" OR "Respiratory Distress Syndrome, Adult" OR "acute respiratory distress" OR "ARDS" OR "ALI" ) AND AB ( "Epidemiology" OR "Incidence" OR "Prevalence" OR "incidence" OR "Mortality" OR "outcome*" OR "ventilator-free day*" OR "length of hospital stay" ) AND AB ( "burn*" OR "thermal injury" ) |
| --- |

**CNKI** search：

| ((SU=烧伤) OR (SU=热烧伤) OR (SU=体表灼伤) OR (SU=皮肤烧伤) OR (SU=体表烧伤)) AND ((SU=急性肺损伤) OR (SU=机械通气性肺损伤) OR (SU=成人呼吸窘迫综合征) OR (SU=急性肺损伤) OR (SU=急性呼吸窘迫) OR (SU=ARDS) OR (SU=ALI)) AND ((FT=回顾性) OR ( FT=流行病学) OR (FT=发生率) OR (FT=患病率)) NOT ((TKA=综述) OR (TKA=进展) OR (TKA="鼠") OR (TKA="小鼠")) |
| --- |

Appendix 2 Quality assessment of the included studies

| **Questions to assess the validity of the study methods and risk of bias** | |
| --- | --- |
| 1. | Is the hypothesis/aim/objective of the study clearly described? |
| 2. | Are the methods for measuring (a) and the main outcomes (b) clearly described? |
| 3. | Is the design of the study described? (a) / Prospective? (b) |
| 4. | Is the setting of the study described? |
| 5. | Is the source of the subjects stated and the sampling frame (population screened) appropriate? (no: mechanically ventilated∙ list of patients with respiratory failure∙) |
| 6. | Is the health outcome measured in an unbiased fashion? (as defined by clinicians; by researcher or trained assessor) |
| 7. | Report the numbers of individuals consistently at each stage of the study? |
| 8. | Are the statistical methods described? |
| 9. | Is the distribution (e.g. by age and gender) of the study population of interest described? |
| 10. | Are the main findings of the study clearly described? |
| 11. | Are any conclusions stated? |
| 12. | Was there ethical approval? |
| 13. | Was there a conflict of interest statement and the role of funding described? |
